# Comprehensive metabolomic study of the response of HK-2 cells to hyperglycemic hypoxic diabetic-like milieu

**DOI:** 10.1038/s41598-021-84590-2

**Published:** 2021-03-03

**Authors:** Alberto Valdés, Francisco J. Lucio-Cazaña, María Castro-Puyana, Coral García-Pastor, Oliver Fiehn, María Luisa Marina

**Affiliations:** 1 Departamento de Química Analítica, Química Física e Ingeniería Química, Universidad de Alcalá, Ctra. Madrid-Barcelona, Km. 33.600, 28871 Alcalá de Henares, Madrid, España; 2 West Coast Metabolomics Center, UC Davis, Davis, CA USA; 3 Departamento de Biología de Sistemas, Universidad de Alcalá, Ctra. Madrid-Barcelona, Km. 33.600, 28871 Alcalá de Henares, Madrid, España; 4 Instituto de Investigación Química Andrés M del Rio, IQAR, Universidad de Alcalá, Ctra. Madrid-Barcelona, Km. 33.600, 28871 Alcalá de Henares, Madrid, España

**Keywords:** Chronic kidney disease, Analytical biochemistry, Mass spectrometry, Metabolomics

## Abstract

Diabetic nephropathy (DN) is the leading cause of chronic kidney disease. Although hyperglycaemia has been determined as the most important risk factor, hypoxia also plays a relevant role in the development of this disease. In this work, a comprehensive metabolomic study of the response of HK-2 cells, a human cell line derived from normal proximal tubular epithelial cells, to hyperglycemic, hypoxic diabetic-like milieu has been performed. Cells simultaneously exposed to high glucose (25 mM) and hypoxia (1% O_2_) were compared to cells in control conditions (5.5 mM glucose/18.6% O_2_) at 48 h. The combination of advanced metabolomic platforms (GC-TOF MS, HILIC- and CSH-QExactive MS/MS), freely available metabolite annotation tools, novel databases and libraries, and stringent cut-off filters allowed the annotation of 733 metabolites intracellularly and 290 compounds in the extracellular medium. Advanced bioinformatics and statistical tools demonstrated that several pathways were significantly altered, including carbohydrate and pentose phosphate pathways, as well as arginine and proline metabolism. Other affected metabolites were found in purine and lipid metabolism, the protection against the osmotic stress and the prevention of the activation of the β-oxidation pathway. Overall, the effects of the combined exposure of HK-cells to high glucose and hypoxia are reasonably compatible with previous in vivo works.

## Introduction

Diabetic nephropathy (DN) is the leading cause of chronic kidney disease and if affects approximately 25–40% of type 1 and type 2 diabetic patients^1,2^. The pathogenesis of DN is characterized by excessive albumin excretion, diabetic glomerular lesions, and loss of glomerular filtration rate^3^. It has been suggested that there is a genetic predisposition to DN, but hyperglycaemia has been determined as the most important risk factor^1^. The increase of glucose plasma levels has been associated with the activation of several metabolic pathways that induce oxidative stress, such as the polyol pathway, the hexosamine flux and the accumulation of advanced glycated end-products (AGEs)^4^. These metabolic changes can also trigger the activation of intracellular second messengers such as protein kinase C (PKC) and MAP kinase (MAPK), nuclear transcription factors such as nuclear factor-κB (NF-κB), growth factors such as the transforming growth factor-β1 (TGF-β1), or the vascular endothelial growth factor (VEGF)^4^.

Over the past two decades, metabolomics technologies have been developed to investigate metabolic changes in different diseases, such as in kidney diseases^5^. Among them, DN changes has been studied in human urine^6^, plasma^7^ and serum^8–10^, rat kidney tissue^11^, mouse urine^12^ and mouse kidney tissue^13^. This has been possible as a result of the technological improvement of separation techniques such as gas chromatography (GC), liquid chromatography (LC) or capillary electrophoresis, as well as the development of robust, high-resolution mass spectrometry (MS) instruments for precise mass determination. However, the broad physicochemical diversity of the metabolome makes essential to combine different analytical platforms to increase the coverage of the identified metabolites^14^. Together with the above-mentioned analytical advances, several bioinformatics tools and processing strategies have been developed to process the large amount of generated analytical data sets^15^. Of special importance is the implementation of tandem mass spectral data (MS/MS) databases for the correct identification of the compounds detected during metabolomic analyses, which are continuously expanding both in coverage and chemical diversity. Another major challenge in the metabolomic field is the biological interpretation of the observed metabolic changes, and several tools and algorithms have been programmed to solve such problems^16–18^.

In DN research it is relevant the study of the proximal tubule, a segment particularly sensitive to hyperglycemia in diabetic conditions that plays a vital role in the pathophysiology of DN^19^. In vitro models of Proximal Tubular Cells (PTC) have demonstrated to be a valuable alternative to animal studies^20^. PTC have been used to explore the mechanisms of DN (including hyperglycaemia, proteinuria, hypoxia and inflammation), being the immortalized HK-2 cell line the most used^20^. Due to their relevance, the high-glucose (HG) induced changes in HK-2 cells have been investigated through different metabolomic platforms, such as capillary electrophoresis-MS^21,22^, hydrophilic interaction liquid chromatography (HILIC)-MS, and reversed-phase liquid chromatography (RPLC)-MS^23^. However, it has been shown that hypoxia also plays a relevant role in the development and progression of DN^24,25^. The combination of hyperglycaemia and hypoxia has been suggested to better mimic of the diabetic-like milieu environment, and the mechanism and consequences of the impaired Hif-1α response to these conditions have been described, highlighting the role of proteasome-dependent mechanisms of HIF-1α degradation^25^. More recently, a time-series study has been performed by our group to evaluate the response to HG-hypoxia at the proteome level, showing that short exposure times do not significantly affect the overall protein expression, but this expression is significantly affected when the treatment is sustained for 48h^26^. However, a comprehensive metabolomic study of the response of HK-2 cells to hyperglycemic, hypoxic diabetic-like milieu has never been performed.

The aim of the present study is to expand the knowledge of the DN progression through a comprehensive metabolomic study in cultured human PTC based on the combination of advanced analytical platforms (GC-time of flight (TOF) MS, HILIC-QExactive (QE) MS/MS and charged-surface hybrid chromatography (CSH)-QE MS/MS). The experimental conditions selected to study the intracellular metabolic changes were the same as in our previous study to complement the information obtained at the protein level^26^, but also including the analysis of the extracellular medium to expand the metabolic picture. Freely available metabolite annotation tools, novel databases and libraries, and stringent cut-off filters were applied to ascertain high quality data. Moreover, advanced bioinformatics and statistical tools were applied to extract information about the most relevant metabolites, chemical classes and metabolic pathways affected. To our knowledge, this is the most comprehensive metabolomics study performed in HK-2 cells subjected to a diabetic-like environment.

## Results

### Protein content

In order to carry out the comprehensive metabolomic study of the changes induced by a diabetic-like hyperglycemic/hypoxic condition in HK-2 cells, five p35 cultured dishes were treated for 48 h with 25 mM glucose (HG)-hypoxic (1% O_2_) conditions and five plates were kept in control (5.5 mM glucose (NG)/18.6% O_2_) conditions. In parallel, the same number of cultured dishes were treated under identical conditions, and the total protein content of the two conditions was measured for proper normalization of the metabolite signals. In total, 70.4 ± 5.51 µg of proteins could be obtained from the control and 60.2 ± 6.42 µg could be obtained from HG-hypoxia, which indicates a decrease of 15% in the protein content after the treatment (*p*-value < 0.05 after two sample t test). These results are similar as those obtained in our previous work, and might correspond to the decrease of 15% on the cell viability observed after 48h^26^. It suggests that the experiment is being performed under similar conditions as before, indicating that the metabolite signals should be corrected by the protein content before any kind of comparison between the conditions studied.

### Metabolite identification

To yield a comprehensive view of intracellular metabolic changes, an untargeted metabolomic analysis on HK-2 cells and extracellular medium was performed. For this aim, three different analytical platforms (GC-TOF MS, HILIC-QE MS/MS, and CSH-QE MS/MS (for lipidomics)) and two different ionization modes (positive and negative), were applied to increase the coverage of metabolites spanning diverse chemical classes (Table 1). Data obtained from each set of samples (cell extracts and extracellular medium), analytical platforms, and ionization modes were firstly processed independently. For each of these platforms, the relative standard deviation of the labelled internal standards included during sample preparation is shown in Supplementary Table S1. The list of all annotated metabolites, MSI annotation level and statistical analyses can be found in Supplementary Tables S2 (intracellular metabolites) and Supplementary Table S3 (extracellular metabolites).

**Table 1:** Number of annotated metabolites in the intracellular and extracellular medium after incubation of HK-2 cells in HG (25 mM glucose)-hypoxia (1% O_2_) and control (5.5 mM glucose/18.6% O_2_) conditions for 48 h and using different analytical platforms and ionization modes.

Platform	Intracellular medium	Extracellular medium
GC-TOF MS	142	81
HILIC-QE MS/MS ( +)	246	168
HILIC-QE MS/MS ( −)	141	92
CSH-QE MS/MS ( +)	194	11
CSH-QE MS/MS ( −)	280	16
Total	733	290

#### Intracellular metabolites

After data processing, the GC-TOF MS analysis of the cell extracts resulted in the annotation of 142 metabolites (Supplementary Table S2). The analysis of the polar fraction by HILIC-QE MS/MS resulted in the annotation of 246 in positive ( +) and 141 in negative (-) modes) in the cell extracts, and the combination of both modes yielded 325 annotated metabolites. Among these metabolites, 60 were commonly annotated in both modes with a good Pearson correlation r values between the retention times and fold change values (r = 0.999 for retention time; r = 0.945 for fold change) (Supplementary Figure S1A and S1B). It has to be noted that even though glucose-1-phosphate and glucose-6-phosphate were annotated in both modes, these metabolites were removed from the analysis because of the bad chromatographic peaks. The analysis of the non-polar fraction by CSH-QE MS/MS resulted in the annotation of 194 in positive ( +) and 280 in negative (-) modes in the cell extracts, giving a total of 355 metabolites. Of these metabolites, 118 were commonly annotated in both modes with a good Pearson correlation r values in both modes (r = 0.992 for retention time; r = 0.893 for fold change) (Supplementary Figure S1C and S1D).

#### Extracellular metabolites

The GC-TOF MS analysis of the extracellular medium resulted in the annotation of 81 metabolites, and the analysis of the polar fraction by HILIC-QE MS/MS resulted in the annotation of 225 metabolites (168 in positive ( +) and 92 in negative (-) modes) (Supplementary Table S3). 34 of these metabolites were annotated in both modes with good Pearson correlation r values in both modes (r = 0.994 for retention time; r = 0.991 for fold change) (Supplementary Figure S2 and S2B). Moreover, the analysis of the non-polar fraction by CSH-QE MS/MS analyses resulted in the annotation of 25 metabolites (11 in positive ( +) and 16 in negative (−) modes), and only 3 metabolites were commonly annotated in both ionization modes.

### Multivariate and univariate analyses

#### Intracellular metabolites

In all platforms (and ionization modes), the principal component analysis (PCA) and the partial least-squares discriminant analysis (PLS-DA) of the intracellular metabolites indicate that the samples are clearly differentiated between the treatments (Supplementary Figures S3 and S4). In GC-TOF MS data, 3 compounds had a VIP > 1.5 (methionine, ornithine and beta-alanine), being all of them with decreased abundance in HG-hypoxia vs control. The univariate analysis of this data yielded 21 and 32 metabolites which abundance was significantly increased and decreased, respectively. The HILIC-QE MS/MS ( +) PLS-DA analysis yielded 22 compounds with VIP > 1.5 (12 increased and 10 decreased), and the HILIC-QE MS/MS (−) yielded 3 compounds with VIP > 1.5 (succinic acid, sorbitol and Ile-Leu), the first two with increased and the last one with decreased values in HG-hypoxia vs control. The univariate analysis of the HILIC-QE MS/MS ( +) data yielded 35 and 9 metabolites significantly increased and decreased, respectively, in HG-hypoxia vs control; and in HILIC-QE MS/MS (−), 19 and 5 metabolites were significantly increased and decreased, respectively. Finally, none of the metabolites had a VIP > 1.5 in the CSH-QE MS/MS ( +) analyses, but 16 compounds had a VIP > 1.5 (all of them with decreased values in HG-hypoxia condition) in CSH-QE MS/MS (−) analyses. The univariate analysis of the CSH-QE MS/MS ( +) data yielded 4 and 9 metabolites with increased and decreased levels, respectively, but zero metabolites were significantly altered in the negative mode.

#### Extracellular metabolites

As it occurs with the intracellular metabolites, the PCA and PLS-DA analyses of the extracellular metabolites clearly differentiated between the conditions studied (Supplementary Figures S5 and S6). In GC-TOF MS, 6 compounds had a VIP > 1.5 (altrose, glucose, lyxose, glucose-1-phosphate, alanine and nonadecanoic acid), all of them with increased values in the HG-hypoxia medium except alanine. The univariate analysis of this data showed 22 and 2 metabolites significantly increased and decreased, respectively, in HG-hypoxia condition. Moreover, the HILIC-QE MS/MS ( +) data analysis yielded 27 compounds with VIP > 1.5 (6 increased and 21 decreased), and in HILIC-QE MS/MS (−), 7 compounds had VIP > 1.5 (6 increased and 1 decreased). The univariate analysis of the positive mode also showed 27 altered metabolites (5 increased and 22 decreased); and 10 and 7 metabolites were significantly increased and decreased, respectively, in negative mode. Finally, the CSH-QE MS/MS analyses (in positive or negative) did not provide any metabolite with VIP > 1.5, but the univariate analysis yielded 2 metabolites (cholesterol ester (CE) 16:1 and sphingomyelin (SM) d40:1) and 6 metabolites (phosphatidylcholine (PC) 36:2, SM d42:2, fatty acid (FA) 22:6; PC 32:1, FA 20:4 and PC 36:1) significantly decreased after the treatment in positive mode and negative modes, respectively.

### Global metabolomic analysis

Data matrices from each platform were combined to generate a joint dataset for intracellular and extracellular metabolites. After the combination and curation, a total of 733 metabolites were annotated in the intracellular medium and 290 in the extracellular medium, in addition to detecting many unknown metabolic signals. Of those annotated metabolites, 65 and 32 were significantly increased and decreased, respectively, in the intracellular medium; and in the extracellular medium, 30 metabolites were increased and 32 were decreased in HG-hypoxia vs control (Supplementary Table S4).

**Figure 1:**
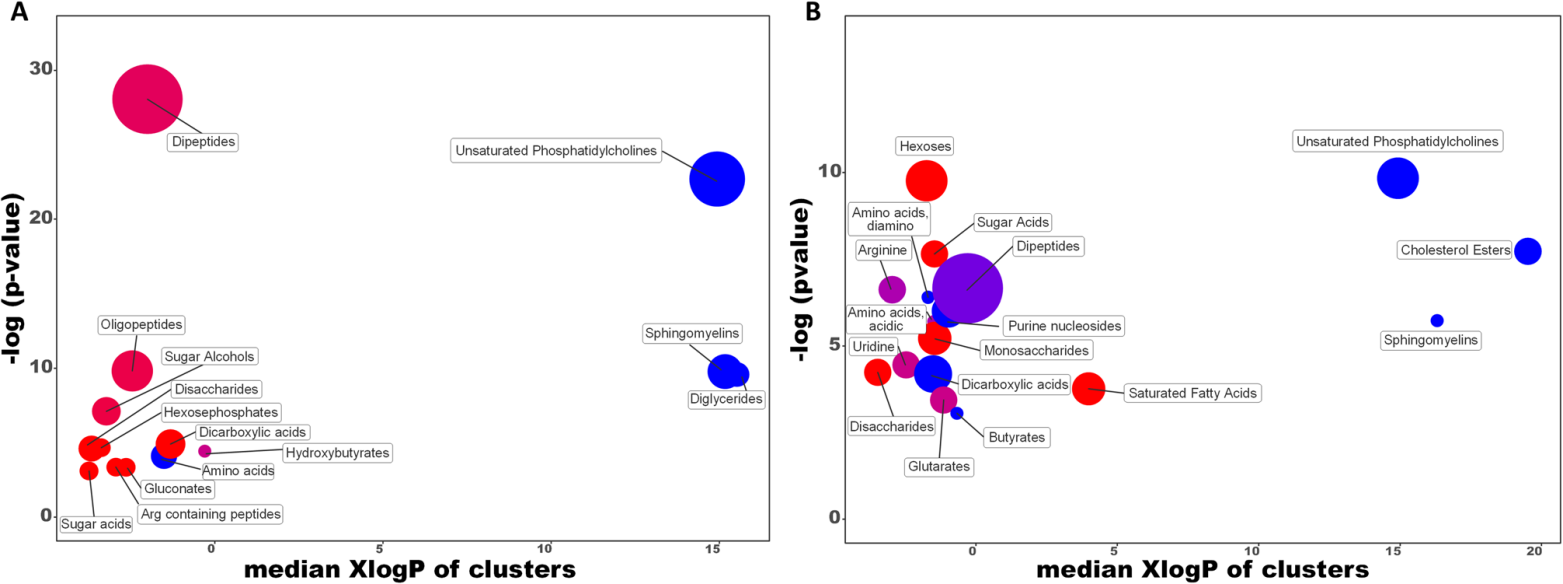
Chemical similarity enrichment analysis of metabolomic data highlighting the differential metabolic regulation in the intracellular medium (**A**) and extracellular medium (**B**) after incubation of HK-2 cells in HG (25 mM glucose)-hypoxia (1% O_2_) compared to control (5.5 mM glucose/18.6% O_2_) conditions for 48 h. Statistical enrichment analysis utilized chemical similarity and ontology mapping to generate metabolite clusters. The y-axis shows most significantly altered clusters on top, x-axis shows XlogP values of metabolite clusters. Cluster colors give the proportion of increased or decreased compounds (red = increased, blue = decreased) in each cluster. Chemical enrichment statistics is calculated by Kolmogorov–Smirnov test. Only enrichment clusters are shown that are significantly different at *p* < 0.05. Plots and calculations were done using ChemRICH.

In order to provide the chemical classes significantly altered in cell extracts and the extracellular medium between the HG-hypoxia vs control, a chemical enrichment analysis using ChemRICH was performed^16^ (Fig. 1). ChemRICH provides enrichment analysis based upon chemical structure and not defined pathways which can be inherently flawed and does not rely upon background databases for statistical calculations^16^. In cell extracts, unsaturated PC, SM, diacylglycerols (DG) and amino acids were significantly decreased while hydroxybutyrates were significantly altered with some species increased, others decreased (Fig. 1A). Dipeptides, oligopeptides, sugar alcohols, dicarboxylic acids, hexosephosphates, disaccharides, Arg containing peptides, gluconates and sugar acids were significantly increased. In the extracellular medium, unsaturated PC and SM were decreased as in the cellular extracts, and CE, amino acids (diamino), purine nucleosides, dicarboxylic acids and butyrates were also decreased (Fig. 1B). Arginine, dipeptides, amino acids (acidic), uridine and glutarates were significantly altered with increased and decreased trends, while hexoses, sugar acids, monosaccharides, saturated FA and disaccharides showed significant increases, this last one similar to the cell extracts.

**Figure 2:**
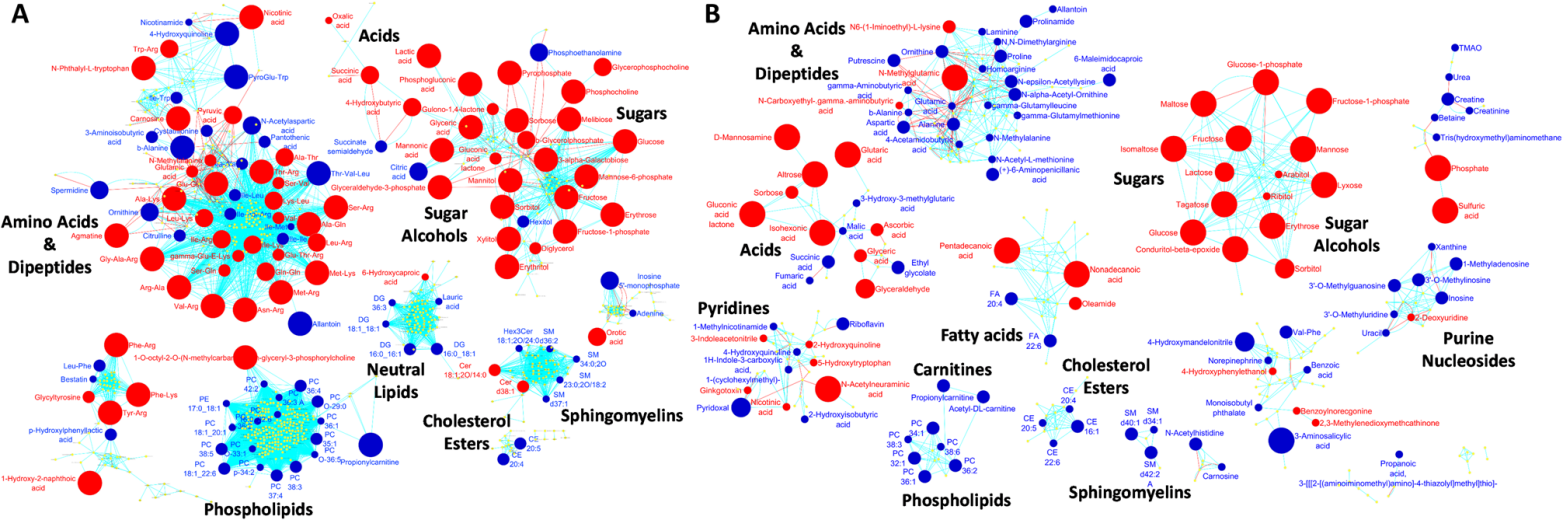
MetaMapp visualization of metabolomic data highlighting the differential metabolic regulation in cell extracts (**A**) and extracellular medium (**B**) after incubation of HK-2 cells in HG (25 mM glucose)-hypoxia (1% O_2_) compared to control (5.5 mM glucose/18.6% O_2_) conditions for 48 h. Red edges denote KEGG reactant pair links and light blue edges symbolize Tanimoto chemical similarity at T > 700. Metabolites found significantly increased (FC > 2) are given as red nodes and blue nodes give decreased metabolites (FC < 0.5). Node sizes reflect fold change. Metabolites that were not found to be differentially regulated are given as yellow nodes and left unlabeled for visual clarity. Significance determined using Mann–Whitney U test with FDR < 0.05.

Additionally, and to highlight the biochemical overrepresentations, all these metabolites were mapped onto biochemical networks constructed using chemical and biochemical similarities from MetaMapp^17^ (Fig. 2). These networks use the KEGG reactant pairs database as foundation for biochemical similarity and Tanimoto substructure composition matrices for chemical similarity mapping. Hence, clusters of compounds visible in these networks represent larger biochemical modules, such as “amino acids & dipeptides”, “sugars” or “phospholipids”. Such modules have the advantage to truly represent all identified metabolites in a data set, while direct pathway mapping (e.g., to KEGG Atlas maps^27^ leaves out many identified metabolites. Overall, the networks showed that the differences in the intra- and extracellular metabolites are not randomly distributed but focused on specific metabolic modules. In both type of samples, the hyperglycemic, hypoxic diabetic-like condition showed a significant increase in sugar and sugar alcohols while phospholipids and CE were decreased. However, the number of altered compounds for these modules was different in each case. Other metabolic modules had opposite direction such as dicarboxylic acids (increased in cell extracts and decreased in the extracellular medium), and some modules were only affected in cell extracts, such as DG (decreased), or in the extracellular medium, such purine nucleosides (decreased).

**Figure 3:**
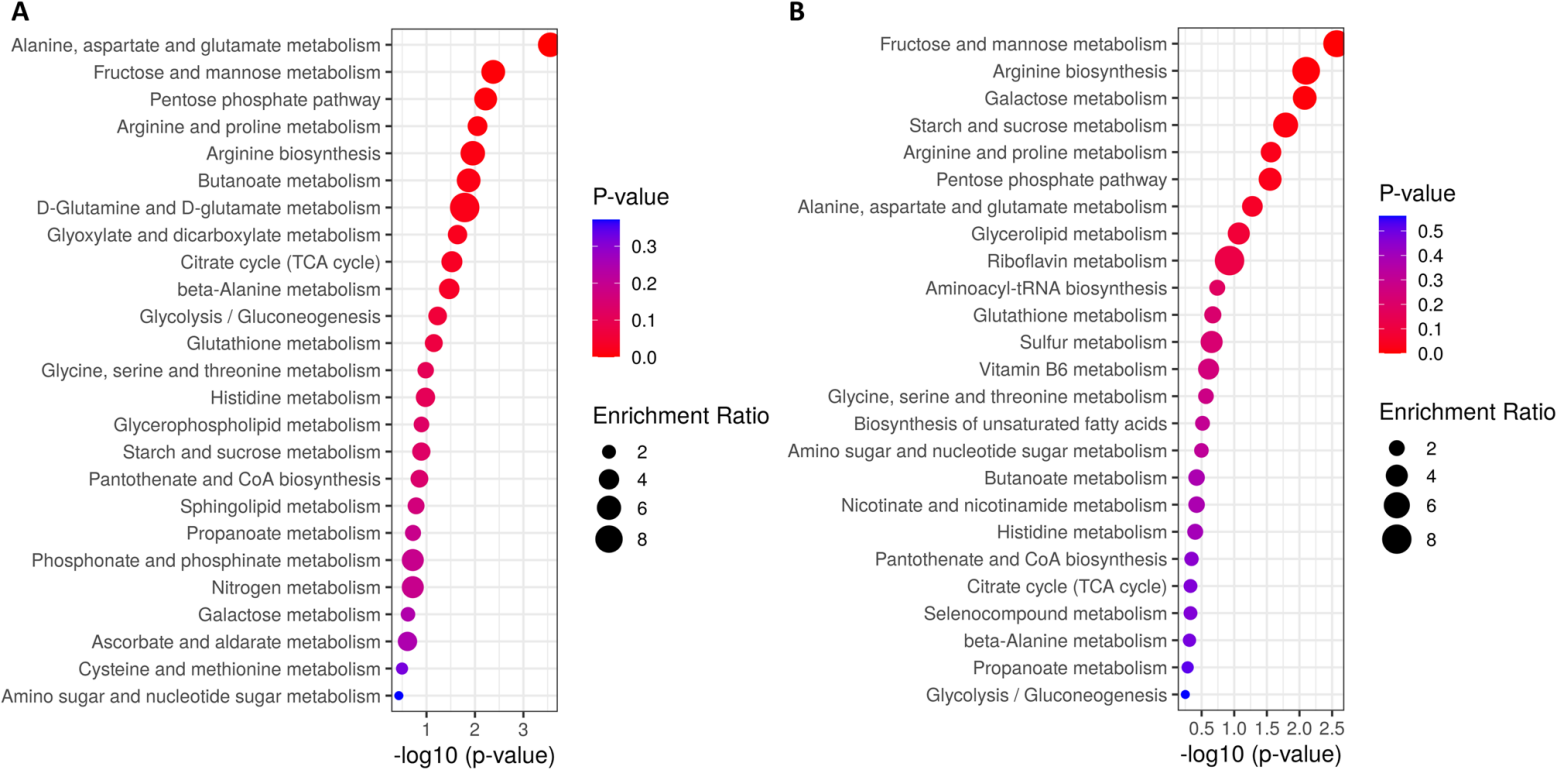
Metabolite Set Enrichment Analysis results from the analysis of metabolites mapped in KEGG human metabolic pathways and significantly altered (after Mann–Whitney U test with FDR < 0.05) in cell extracts (**A**) and extracellular medium (**B**) after incubation of HK-2 cells in HG (25 mM glucose)-hypoxia (1% O_2_) compared to control (5.5 mM glucose/18.6% O_2_) conditions for 48 h.

Finally, metabolite set enrichment analysis was performed using MetaboAnalyst 4.0^18^ (Fig. 3). When analysing the intracellular metabolites, it has to be noted that only 57 out of the 97 altered metabolites could be mapped with valid KEGG IDs (most dipeptides and some PC could not be mapped). This analysis showed that the *Alanine, aspartate and glutamate metabolism* was the most significantly enriched metabolite set, with 6 metabolites matching to this pathway (3 with increased levels: glutamate, pyruvate and succinate; and 3 with decreased levels: citrate, N-acetyl-l-aspartate, succinate semialdehyde) (Fig. 3A). The second most enriched set was the *Fructose and Mannose metabolism* (with sorbitol, mannose 6-phosphate, fructose and fructose 1-phosphate more abundant in HG-hypoxia vs control), followed by the *Pentose phosphate pathway* (with glyceraldehyde 3-phosphate, 6-phosphogluconic acid, gluconolactone and glyceric acid with increased levels). The last enriched metabolite set with a *p*-value < 0.01 was the *Arginine and proline metabolism*, with 5 altered metabolites (3 increased: agmatine, glutamate and pyruvate; and 2 decreased: spermidine and ornithine). When analysing the extracellular metabolites, 47 out of the 62 altered metabolites could be mapped with valid KEGG IDs. The enrichment analysis showed 3 metabolite sets with *p*-value < 0.01: *Fructose and mannose metabolism* (with sorbitol, fructose, mannose and glyceraldehyde increased in HG-hypoxia vs control); *Arginine biosynthesis* (with N-acetylornithine, aspartic acid and ornithine decreased); and *Galactose metabolism* (with lactose, fructose, mannose and sorbitol with increased levels) (Fig. 3B).

### Integration of metabolomic and proteomic data

To complement the metabolomics results, the 57 intracellular altered metabolites were combined with the 27 altered proteins (observed in our previous study^26^) and analysed together using the Joint Pathway Analysis from MetaboAnalyst 4.0. This analysis provided a total of 4 significantly enriched pathways (Table 2), some of them already observed as enriched when using the Metabolite Set Enrichment Analysis.

**Table 2:** Significantly enriched KEGG human metabolic pathways from the analysis of significantly altered metabolites (after Mann–Whitney U test with FDR < 0.05) and significantly altered proteins (from Valdés et al. 2020^26^) after incubation of HK-2 cells in HG (25 mM glucose)-hypoxia (1% O_2_) and control (5.5 mM glucose/18.6% O_2_) conditions for 48 h.

KEGG pathway	Total	Hits	Metabolites/proteins	Raw p	− log(*p*)	FDR	Impact
Glycolysis or Gluconeogenesis	61	12	4/8	2.97E−10	9.527	2.49E−08	0.9667
Fructose and mannose metabolism	40	7	4/3	5.30E−06	5.276	2.23E−04	0.8974
Pentose phosphate pathway	47	6	4/2	1.70E−04	3.769	4.77E−03	0.6087
Alanine, aspartate and glutamate metabolism	61	6	6	7.25E−04	3.140	1.52E−02	0.3333

Among these pathways, *Glycolysis or gluconeogenesis* pathway was the most enriched, with 12 hits: 4 metabolites (pyruvate, lactate, glyceraldehyde 3-phosphate and glucose) and 8 proteins (LDHA, PKM, PGAM4, GAPDH, TPI1, GPI, PGK1 and ALDOA). The second most enriched pathway was the *Fructose and mannose metabolism*, with 7 hits: 4 metabolites (sorbitol, mannose 6-phosphate, glyceraldehyde 3-phosphate and fructose 1-phosphate) and 3 proteins (AKR1B1, TPI1 and ALDOA). Another enriched pathway was the *Pentose phosphate pathway*, with 6 hits: 4 metabolites (glyceraldehyde 3-phosphate, 6-phospho-gluconate, glucono-1,5-lactone and glycerate) and 2 proteins (GPI and ALDOA). In the three pathways, all the metabolites and proteins were significantly increased, suggesting their activation. The last enriched pathway was the *Alanine, aspartate and glutamate metabolism* pathway, considered by the different abundance of 6 metabolites: N-acetyl-L-aspartate, succinate semialdehyde and citrate, with decreased levels; and L-glutamate, pyruvate and succinic acid, with increased levels in HG-hypoxia vs control.

## Discussion

The role of proximal tubule in the development of DN has been widely investigated in the last years, and several hallmarks have been defined as contributing to the progression of this disease^4,19^. The increase of glucose plasma levels and the associated metabolic changes in the proximal tubule have been established as the most important factors, and several metabolomic studies using in vivo and in vitro models have been performed to unravel the mechanisms and consequences involved^5^. In the present study, and to expand the knowledge on the DN research, a comprehensive metabolomic study has been performed to investigate the metabolic response of HK-2 cells to hyperglycemic, hypoxic diabetic-like milieu conditions. The experimental conditions selected were the same as in previous studies^25,26^, and the analysis of the extracellular medium was also performed. As it can be observed in the results section, the combination of different separation techniques (GC, HILIC and CSH, for lipidomics) coupled to high resolution MS (TOF and QE), the use of different ionization modes, and the application of advanced bioinformatics tools, freely available metabolite annotation tools, novel databases and libraries, and stringent cut-off filters allowed the annotation of more than 730 metabolites in the cell extracts and 290 metabolites in the extracellular media. This is, as far as the author’s knowledge, the most comprehensive study performed in HK-2 cells in the DN research field. Moreover, advanced bioinformatics and statistical tools such as MetaboAnalyst, ChemRich and MetaMapp have been applied to extract information about the most relevant metabolites, chemical classes and metabolic pathways affected. In our previous proteomics work we observed that the most significant changes occurred after 48 h of HG-hypoxia treatment, and several proteins involved in the *glycolytic pathway*, were observed as up-regulated (LDHA, PKM, PGAM4, GAPDH, TPI1, GPI, PGK1 and ALDOA)^26^. The current metabolomic study complements these results, with four intracellular metabolites involved in this pathway observed as increased (pyruvate, lactate, glyceraldehyde 3-phosphate and glucose). Glucose was also more abundant in the extracellular medium (due to the used of HG medium), and other sugars were also significantly increased (fructose, mannose, lactose, maltose, isomaltose, sorbose, tagatose, erythrose and lyxose). It is known that PTC are gluconeogenic in vivo^28^ and that they use mainly oxygen for aerobic respiration under physiological condition (although they also show reasonable amounts of glycolysis^29^), so that PTC metabolize mainly fatty acids, glutamine and lactate to produce energy and reduced nicotinamine adenine dinucleotide necessary to maintain the functions of the proximal tubule^28,30^. Although HK-2 cells have glucogenic activity^31,32^, they also have high rates of glycolysis in basal conditions^33,34^ (as many other types of cultured PTC), which is a potential limitation of our study. But, given that proximal tubule glycolytic flux plays an important pathogenic role in DN^35^, the increase found by us in metabolites involved in glycolysis might be pathologically relevant. A previous metabolomics study using HK-2 cells treated with 25 mM glucose for 24 h has shown that the lactate to pyruvate ratio is increased and that some TCA cycle metabolites are decreased (citric acid, α-ketoglutaric acid and fumaric acid)^21^. Our data agree well with those results as we have shown that the levels of lactate are more increased (FC > 18) that the levels of pyruvate (FC > 3), and that the levels of citric acid are significantly reduced (FC = 0.27). On the other hand, pyruvate, lactate and glyceraldehyde 3-phosphate have been also found increased in both urine^36^ and renal cortex samples (the kidney cortex is ≈ 90% proximal tubules)^35^ from db/db mice, a representative animal model for DN. Succinic acid is also involved in the gamma-hydroxybutiric acid metabolism. The gamma-hydroxybutyric acid (4-hydroxybutyric acid) is found in the mammalian brain, heart, liver, and kidneys. Under physiological conditions, it is metabolized to succinic semialdehyde by 4-hydroxybutyrate dehydrogenase and then transformed to succinic acid^37^ by succinic semialdehyde dehydrogenase. In the present study, we observed the levels of 4-hydroxybutyric acid as increased and succinate semialdehyde as decreased, which could also contribute to the increased levels of succinic acid observed. However, none of the enzymes involved in this transformation were identified in our previous proteomics work^26^, highlighting the importance of metabolomics studies to expand or complement the information obtained from other high-throughput technologies (such as proteomics) for a better understanding of disease processes.

Some of the enzymes and metabolites involved in the glycolysis process are also part of the *polyol pathway*. In our recent proteomic study, the rate-limiting enzyme AKR1B1 implicated in the reduction of glucose to sorbitol was observed as up-regulated at 48h^26^. It has been suggested that the polyol pathways is altered in DN^38,39^, however this pathway is not considered in KEGG database. Our metabolomic findings indicate that sorbitol levels are highly increased, as well as the levels of fructose, fructose 1-phosphate and glyceraldehyde 3-phosphate, which in combination with the up-regulation of GPI, GAPDH, TPI1 and ALDOA suggest the activation of the polyol pathway. The activation of this pathway has been related with the depletion of ATP in proximal tubular cells^38^, and in our previous proteomics findings we observed the down-regulation of the ADP/ATP Translocase 2 (SLC25A5) and the ATP Synthase F1 Subunit Beta (ATP5B), which have been shown to decrease the levels of ATP in the cytosol and to aggravate DN^40,41^. The reduction of glucose to sorbitol has also been related with decreased levels of NADPH and NAD + , preventing their use in important pathways such as glutathione and nitric oxide (NO) production^42^. To regenerate NADPH and NAD + , the pentose phosphate pathway is usually activated. As observed in the metabolite set enrichment analysis and the joint pathway analysis, the pentose phosphate pathway was overrepresented in the intracellular medium, based on the increased levels of glyceraldehyde 3-phosphate, 6-phospho-gluconate, glucono-1,5-lactone and glycerate metabolites. In addition, the levels of nicotinic acid (a precursor of the NAD +) were also found increased, and all these results suggest that this pathway is activated. Overactivation of the pentose phosphate pathway has been previously found in kidneys from streptozotocin-induced diabetic mice^43^ and it might be a potentially protective mechanism in hyperglycaemia due to shunting of glycolytic intermediates into pentose phosphate pathway reactions^44^. The activation of the polyol pathway can also generate AGEs intermediates that can glycosylate protein residues. In this sense, mannose is the major monosaccharide component of N-glycans^45^, and as shown in the results section, the fructose and mannose metabolism pathway was observed overrepresented mainly because of the increased abundance of mannose 6-phosphate. In addition, some of the metabolites involved in this pathway were observed as increased in the extracellular medium (sorbitol, fructose, mannose and glyceraldehyde), suggesting that these metabolites are excreted by the cells.

Apart from the activation of glycolysis and the polyol pathway, another broadly described mechanism of pathogenesis in DN is the activation of the PKC signalling^46,47^. PKC is activated by DG that are formed from excess of glyceraldehyde-3-phosphate during diabetes. However, the chemical enrichment analysis performed indicates that several DG are decreased (DG 34:1, DG 36:1, DG 34:2, DG 34:3, DG 36:2, DG 36:3 and DG 32:1), being the last one significantly decreased. Different cell culture studies have demonstrated that hypoxia conditions or increasing glucose levels in the media can elevate the cellular levels of DG^46,48^, and that there is a close correlation between DG production and HIF-1α expression upon hypoxic stimulation^48^. We have previously demonstrated the proteasome-dependent mechanisms of HIF-1α degradation under HG and hypoxia conditions^25^, which could be related with the degradation of DG. However, further studies are needed to validate this hypothesis.

The *arginine and proline metabolism pathway* was also overrepresented in the intracellular medium due to the alteration of ornithine, spermidine, agmatine, glutamate and pyruvate metabolites. Arginine is a semi essential amino acid synthesised from glutamine, glutamate and proline, and its degradation occurs via multiple pathways^49^. One of this degradation pathways is the transformation of arginine to urea and ornithine, being the last one the precursor for the synthesis of polyamines (putrescine, spermidine and spermine). As shown in Supplementary Table S4, the levels of ornithine and spermidine were significantly decreased in the intracellular medium, and the levels of ornithine, N-acetylornithine and putrescine were less abundant in the extracellular medium. Furthermore, arginine can be metabolized to NO and citrulline by nitric oxide synthethase (NOS) in the cytosol; or arginine can be synthesized from citrulline through the arginino succinate pathway. It has been reported that the NO production is decreased in patients with chronic kidney disease^50^, and our results indicate that the levels of citrulline are decreased in the intracellular medium, suggesting that arginine is not being degraded to citrulline. Moreover, arginine can me metabolized to agmatine in the mitochondria by the arginine decarboxylase (ADC). It has been suggested that agmatine can inhibit the activity of ornithine decarboxylase (ODC) with a decrease of the synthesis of polyamine metabolites^51^; it can regulate the activity of NOS isoenzymes^52^; or it can reduce the collagen accumulation in kidneys of diabetic mice^53^. In our study, the levels of agmatine were found increased, and according to those references, these levels could explain the decreased levels of polyamines and citrulline observed. The chemical similarity enrichment analysis and the Mann–Whitney U test analysis also shown that the levels of several arginine-containing dipeptides are increased in the intracellular medium (Arg-Ala, Asn-Arg, Glu-Thr-Arg, Gly-Ala-Arg, Ile-Arg, Leu-Arg, Met-Arg, Phe-Arg, Ser-Arg, Thr-Arg, Trp-Arg, Tyr-Arg, Val-Arg). Their appearance could be due to protein catabolism to obtain free arginine, or they could be used for de novo synthesis of proteins. The observed decrease of 15% in the total protein amounts after the treatment in this and in our previous work^26^ suggests that the proteins are being degraded, but further studies are needed to verify this hypothesis. Altogether, and despite of the above-mentioned altered metabolites, arginine levels were not affected in the intracellular nor in the extracellular medium, suggesting that arginine metabolism is highly controlled in the conditions studied.

Of special importance is the increased abundance of carnosine, a dipeptide made up of the amino acids beta-alanine and histidine. Beta-alanine is the rate-limiting precursor for the synthesis of carnosine and it was observed decreased after the treatment. Carnosine has been shown to improve diabetes and glucose metabolism in BTBR *ob/ob* mice^54^. In addition, carnosine inhibits the increased production of fibronectin and collagen type VI in podocytes and the increased production of TGF-β in mesangial cells induced by 25 mM glucose^55^. This result may also explain the down-regulation of fibronectin protein observed in our previous proteomic study^26^.

Two interesting metabolites with increased levels in the cell extracts are glycerophosphorylcholine and phosphocholine, the two major forms of choline storage in the cytosol. Glycerophosphocholine has been found in high abundance in renal tissue, and this and other organic compounds (such as sugar alcohols) are considered osmolytes that can protect renal medullary cells from high concentrations of NaCl and urea^56^. However, osmotic pressure can also be caused by HG levels, and apart from glycerophosphocholine, the sugar alcohols sorbitol, xylitol, erythritol and mannitol were found increased in the intracellular medium. Moreover, it has been described that glycerophosphocholine is synthesized from phosphatidylcholine and broken down into choline and glycerol-3-phosphate^56^, and as shown in the chemical enrichment analysis, the levels of unsaturated PC were decreased with several of them significantly altered (PC 40:7, PC 35:1, PC 38:5 and PC 36:4). On the other hand, phosphatidylcholine is the major phospholipid in cell membranes. Its main function, in collaboration with other phospholipids and cholesterol, is to maintain the structure of cellular membranes. Phosphatidylcholine also has regulatory roles in cells and it participates in cellular signalling^57^ so that abnormal cellular phosphatidylcholine to phosphatidylethanolamine molar ratios can influence energy metabolism in various organelles and have been linked to disease progression^58^.

Diabetic-milieu also induced other changes in the lipid profile in HK-2 cells: there was an increase in ceramides (Cer 32:1 and Cer 38:1) and a decrease in cholesterol esters (CE 20:4 and CE 20:5). Ceramides are bioactive lipids that play important roles in many cellular processes such are growth, differentiation and apoptosis. It has been previously shown that ceramides generated by ceramide synthase activation induce renal proximal tubular cell injury in response to hydrogen peroxide^59^ or hypoxia/reperfusion^60^. Regarding cholesterol, it exists in a ≈ 1:5 ratio with phospholipid content in plasma membranes from renal tubular cells. Hypoxic and oxidant injury each induce ≈ 33% decrements in cholesterol esterified levels in isolated mouse proximal tubule segments, this resulting in ≈ 50 to 60% LDH release^61^. When the decline in cholesterol esterified is induced by other means, acute cytotoxicity also results^62,63^. In summary, our results open the possibility that changes in the lipid profile in proximal tubules similar to those found in HK-2 cells exposed to the diabetic-like milieu might contribute to the progressive loss of viability of proximal tubular cells found in a significant proportion of diabetic patients^64^. However, specific experiments should be performed to confirm this hypothesis.

Another interesting metabolite found decreased is the inosine 5′monophosphate (IMP). IMP is synthesized from ribose 5-phosphate and can be oxidized to xanthosine monophosphate, a key precursor in purine metabolism. Adenine and guanine are derived from IMP, and several purine metabolites involved in the salvage pathway were found with increased values but with FDR p-values close to 0.05: 2′-O-methylinosine, 3′-O-methylinosine, 3′-O-methylguanosine, guanosine, ribose-5-phosphate, adenosine-5-monophosphate and allopurinol (see Supplementary Table S2). However, the abundance of allantoin (the last product of purine degradation) was observed as decreased suggesting that the synthesis and not the degradation of purine metabolites is being performed. The decreased levels of this metabolite agrees with a previous study where the urine levels of allantoin are decreased in an age-dependent manner in *db/db* mice^13^. Moreover, the orotic acid involved in the biosynthetic pathway of pyrimidines was also observed as increased in the intracellular medium, and it has been related with beta-alanine release and increasing levels of carnosine^65^. On the contrary, several purine metabolites were found decreased in the extracellular medium (inosine, 1-methyladenosine, 3′-O-methylinosine and 3′-O-methylguanosine), indicating that purine metabolism is important in the development of DN.

Finally, one of the most significantly altered (decreased) metabolite in the intracellular medium was propionyl-L-carnitine, which was also less abundant in the extracellular medium in HG-hypoxia vs control (as well as acetyl-L-carnitine). Acyl-carnitines are short chain fatty acid esterified to carnitine that are rapidly transported into cells where they are transformed into free carnitine and propionyl (or acetyl) coenzyme A for energy supply^66^. Acyl carnitines have been previously associated with the progression of DN in urine samples of diabetic patients^6,35^. Another metabolite less abundant in the intracellular medium and that has been related with the fatty acid β-oxidation is 3-aminoisobutyric acid (or β-aminoisobutyric acid), and this metabolite has been inversely associated with insulin secretory function in humans^67^.

It should be noted that our work has several limitations. In the first place, it is necessary to take into account the shortcomings of cultured HK-2 cells as a model of the proximal tubule: HK-2 cells, as well as many of the available proximal tubule model cell lines fail to replicate the differential expression of several uptake and efflux membrane transporters and metabolizing enzymes, which is one of the characteristics of native PTC^68^. In addition, as indicated above, HK-2 cells and other proximal tubule cell lines have higher rates of glycolysis than native proximal tubules. On the other hand, PTC display metabolic zonation, with different enzyme machineries and transporters along proximal tubule segments S1, S2 and S3^69^ and HK-2 cells might be representative of only one of them. For instance, they express monocarboxylate transporter MCT1^70^, which is restricted to the S1 segment^71^. Cultured PTC are the most widely used tool for in vitro proximal tubule research and HK-2 cells represent particularly well regarding the effects of diabetes on proximal tubules: around 50% of the articles published in the field in the last two years involve studies in HK-2 cells (results from a PubMed search). Therefore, a complete understanding of HK-2 cell metabolism will contribute to better decide how and when to use them in research and which consideration should be taken for the best interpretation of the data. This will help to answer very relevant questions such are whether HK-2 cells a model of a specific segment of the proximal tubule or whether the differences with native renal PTC are just the consequence of the immortalization and culture conditions of HK-2 cells. Metabolomic studies in 3D cell culture models, particularly these involving proximal tubule-on-a-chip technolology^68^, or proximal tubule cell lines established from human urine^72,73^, are other alternative promising strategies to understand the metabolic changes induced by diabetes in PTC and their contribution to DN.

In summary, the comprehensive metabolomic study performed using a PTC model subjected to HG-hypoxia confirms the alteration of several intracellular metabolites and metabolic pathways already known to be affected in DN, and also suggests some other metabolites to be investigated in future works for a better understanding of this disease. However, it has also shown some limitations when compared with in vivo studies in which native PTC are exposed to the real diabetic environment.

## Conclusion

This is the most comprehensive metabolomic study based on a combination of different metabolomic platforms (GC-TOF MS, HILIC-QE MS/MS and CSH-QE MS/MS) together with advanced bioinformatics and statistical tools carried out to analyse the effects of the diabetic milieu on in vitro PTC using a very simplified model of the diabetic microambient (i.e. HG and hypoxia). The results obtained in this study demonstrate that the combined exposure to HG and hypoxia reproduces most of the effects observed in vivo as several pathways (glucose, polyol and pentose phosphate pathways), as well as the arginine and proline metabolism are altered. Other significantly affected metabolites could be related with the alteration of the purine and lipid metabolism, the protection against the osmotic stress or the prevention of the activation of the β-oxidation pathway. However, the utilized model also has some limitations.

## Methods

### Chemicals and reagents

LC–MS-grade water solvents (acetonitrile (ACN) and methanol) were obtained from Fisher Scientific (Waltham, MA, USA) and isopropanol, formic acid, ammonium formate, ammonium acetate, methyl tert-butyl ether (MTBE), and toluene were purchased from Sigma-Aldrich/Fluka (St. Louis, MO, USA). The internal standard 12-[[(cyclohexylamino)-carbonyl]amino]-dodecanoic acid (CUDA) was purchased from Cayman Chemical (Ann Arbor, MI, USA) and CE 22:1 was obtained from Nu-Chek (Elysian, MN, USA). The lipid standards lysophosphatidylethanolamine (LPE) 17:1, phosphoinositol (PI) 15:0/18:1-d_7_, phosphatidylserine (PS) 15:0/18:1-d_7_, lysophosphatidylcholine (LPC) 17:0, PC 12:0/13:0, phosphatidylethanolamine (PE) 17:0/17:0, phosphatidylglycerol (PG) 17:0/17:0, cholesterol-d_7_, SM d18:1/17:0, ceramide (Cer) d18:1/17:0, sphingosine (d17:1), monoacylglycerol (MG) 17:0/0:0/0:0, DG 12:0/12:0/0:0 and 18:1/2:0/0:0, triacylglycerols (TG) 17:0/17:1/17:0-d_5_ and 14:0/16:1/14:0-d_5_, and 5-[((13,13,14,14,15,15,16,16,16-d_9_)palmitoyl)hydroxy]-stearic acid (5-PAHSA) were provided by Avanti Polar Lipids (Alabaster, AL, USA). The isotope labelled standard palmitic acid-d_3_, creatinine-d_3_ (methyl-d_3_), d_9_-betaine.HCl, Nτ-methyl-d_3_-histamine.2HCl, d_3_-creatine.H2O (methyl-d_3_), DL-alanine-3,3,3-d_3_, L-glutamine-2,3,3,4,4-d_5_, DL-glutamic-2,4,4-d_3_ acid and DL-aspartic-2,3,3-d_3_ acid were purchased from CDN Isotopes Inc. (Pointe-Claire, QC, CAN). d_9_-choline chloride, trimethylamine N-oxide (TMAO)-d_9_, d_3_-L-carnitine.HCl, 15N_2_-L-arginine.HCl, DL-cystine (3,3,3′,3′-D_4_), L-alanine (2,3,3,3,-d_4_), L-arginine:HCL-d_7_, L-asparagine:H20 (2,3,3-d_3_), L-glutamic acid (2,3,3,4,4-d_5_), L-histidine:HCL:H20-d_5_, L-isoleucine-d_10_, L-leucine-d_10_, L-lysine:2HCl (3,3,4,4,5,5,6,6-d_8_), L-methionine-d_8_, L-ornithine:HCL-d_2_, L-phenylalanine-d_8_, L-proline-d_7_, L-serine (2,3,3-d_3_), L-threonine-d_5_, L-tryptophan-d_8_, L-tyrosine-d_7_, and L-valine-d_8_ were obtained from Cambridge Isotope Laboratories Inc. (Andover, MA, USA), 1-methylnicotinamide-d_3_ Iodide was purchased from Toronto Research Chemical (Toronto, ON, Canada), and Val-Tyr-Val and [d_3_]acetyl-L-carnitine.HCl were provided by Sigma-Aldrich (St. Louis, MO, USA).

### HK-2 Cell culture conditions

Human proximal tubular epithelial (HK-2) cells were obtained from the American Type Culture Collection (Rockville, MD, USA), and they were cultured in the same conditions as previously described^26^. Briefly, DMEM/F12 (ThermoFisher, Grand Island, NY, USA) supplemented with 10% FBS, 1% penicillin/streptomycin/amphoterycin B, 1% glutamine and 1% insulin-transferrine-selenium (ITS) (ThermoFisher, Grand Island, NY, USA) was used to grow the cells, and this medium was changed to DMEM normal glucose (NG, 5.5 mM glucose, ThermoFisher, Grand Island, NY, USA), with the same supplementation as described above, one week before the beginning of the experiments. To perform the experiments, P35 cultured plates were seeded with 2.5 × 10^5^ cells, and cells were exposed to either medium DMEM NG or DMEM high glucose (HG, 25 mM glucose, ThermoFisher, Grand Island, NY, USA) supplemented with 0.5% FBS, 1% penicillin/streptomycin/amphoterycin B, 1% glutamine and 1% ITS under hypoxic (1% O_2_) or control conditions (18.6% O_2_) for 48 h (cell culture medium was refreshed after 24 h). A total of ten different P35 replicates were used for each condition, where five of these plates were employed for the metabolomics study and the other five were used for total protein measurement as previously describe^26^.

For the intracellular metabolite analysis, cells were washed three times with PBS, trypsinized, and washed again with PBS. Finally, they were centrifuged at 2500 rpm for 5 min and cell pellets were stored at − 80 °C until sample preparation. The extracellular medium of each condition was collected and stored at − 80 °C until sample preparation.

### Metabolite extraction

Extraction of intracellular and extracellular metabolites was carried out using a biphasic solvent system consisting on cold methanol, MTBE, and water^74^ with some modifications. In more detail, 225 μL of methanol at − 20 °C containing an internal standard mixture of LPE (17:1), PI (15:0/18:1)-d_7_, PS (15:0/18:1)-d_7_, LPC (17:0), PC (12:0/13:0), PE (17:0/17:0), PG (17:0/17:0), cholesterol-d_7_, SM (d18:1/17:0), Cer (d18:1/17:0), sphingosine (d17:1), MG (17:0/0:0/0:0), DG (12:0/12:0/0:0), DG (18:1/2:0/0:0), TG (17:0/17:1/17:0)-d_5_, TG (14:0/16:1/14:0)-d_5_, palmitic acid-d_3_, and 5-PAHSA-d_9_ was added to the cell pellets or to 20 μL of extracellular medium. Thereafter, 750 μL of MTBE at − 20 °C containing CE (22:1) was added to the samples (the concentration of each internal standard can be found in Supplementary Table S5). In the case of cell pellets, the samples were ground using a GenoGrinder 2010 (SPEX SamplePrep) for 2 min at 1350 rpm and in the case of extracellular medium, samples were shaken for 6 min at 4 °C with an Orbital Mixing Chilling/Heating Plate (Torrey Pines Scientific Instruments). Then phase separation was induced by adding 250 μL of LC–MS-grade water and vortexed, followed by centrifugation at 14,000 rpm for 2 min. 350 μL of the upper layer was collected for the analysis of non-polar compounds using LC–MS, and 125 μL of the bottom layer was collected twice for the analysis of polar compounds using LC–MS and GC–MS. Thereafter, every fraction was evaporated to dryness.

### Gas chromatography-time-of-flight mass spectrometry (GC-TOF MS) analysis

The first dried polar fractions of the cell extracts and the extracellular medium were derivatized and analysed as previously described^75^. Derivatization was performed by shaking the samples at 30 °C for 90 min with 10 μL of methoxyamine hydrochloride in pyridine (40 mg/mL), followed by trimethylsilylation at 37 °C for 30 min with 90 μL of N-methyl-N-(trimethylsilyl) trifluoroacetamide (MSTFA, Sigma-Aldrich) containing C8-C30 fatty acid methyl esters (FAMEs) as internal standards (their concentration can be found in Supplementary Table S5). Aliquots of 0.5 μL of derivatized samples were analyzed using a Leco Pegasus IV time-of-flight (TOF) MS (Leco Corporation) coupled to an Agilent 6890 GC (Agilent Technologies) equipped with a 30 m long 0.25 mm id Rtx-5Sil MS column (0.25 μm film thickness) and a Gerstel MPS2 automatic liner exchange system (Gerstel GMBH & Co. KG). The chromatographic gradient used a constant flow of 1 mL/min with following gradient: 50 °C (1 min), 20 °C/min to 330 °C, hold 5 min. Mass spectrometry data was collected using 1525 V detector voltage at m/z 85–500 with 17 spectra/s, electron ionization at − 70 eV and an ion source temperature of 250 °C. QC injections, method blanks and a pooled mixture of all control and treated samples (cells extracts and extracellular medium separately) were included as quality control samples.

### Hydrophilic interaction liquid chromatography-QExactive mass spectrometry (HILIC-QE MS/MS) analysis

The second dried polar fractions from cell extracts or the extracellular medium were resuspended in 50 μL of ACN:water (4:1, v/v) mixture containing 10 μg/mL of CUDA and a mixture of internal standards compounds (creatinine-d_3,_ betaine-d_9_, Nτ-methyl-histamine-d_3_, creatine-d_3_, DL-alanine-d_3_, L-glutamine-d_5_, DL-glutamic acid-d_3_, DL-aspartic acid-d_3_, choline-d_9_, TMAO-d_9_, L-carnitine-d_3_, 15N_2_-L-arginine, DL-cystine-d_4_, L-alanine-d_4_, L-arginine-d_7_, L-asparagine-d_3_, L-glutamic acid-d_5_, L-histidine-d_5_, L-isoleucine-d_10_, L-leucine-d_10_, L-lysine-d_8_, L-methionine-d_8_, L-ornithine-d_2_, L-phenylalanine-d_8_, L-proline-d_7_, L-serine-d_3_, L-threonine-d_5_, L-tryptophan-d_8_, L-tyrosine-d_7_, L-valine-d_8_, 1-methylnicotinamide-d_3_, Val-Tyr-Val and acetyl-L-carnitine-d_3_) (their concentration is shown in Supplementary Table S5). In the case of cell extracts, 10 μL of samples were diluted (1:4) with the resuspension solvent to avoid column MS signal saturation. Aliquots of 3 μL (for both ESI positive and ESI negative) were injected into a LC–MS/MS system consisting of an Vanquish UltiMate 3000 quaternary UHPLC (Thermo Fisher Scientific, Bremen, Germany) coupled to a QExactive HF Mass Spectrometer (Thermo Fisher Scientific, Bremen, Germany). Compounds were separated using a Waters Acquity UPLC BEH Amide column (150 mm length × 2.1 mm id; 1.7 μm particle size) with an additional Waters Acquity VanGuard BEH Amide pre-column (5 mm × 2.1 mm id; 1.7 μm particle size). Columns were kept at 45◦C during the analysis of the samples. The separations were performed at a flow rate of 0.4 mL/min with mobile phases A (100% (v/v) water, 10 mM ammonium formate and 0.125% (v/v) FA) and B (95% (v/v) ACN, 10 mM ammonium formate and 0.125% (v/v) FA). Gradient elution was performed from 100% (B) at 0–2 min to 70% (B) at 7.7 min, 40% (B) at 9.5 min, 30% (B) at 10.25 min, 100% (B) at 12.75 min, isocratic until 16.75 min. The mass spectrometer was coupled using a HESI-II electrospray ionization source with the following parameters: spray voltage, + 3 kV in positive and − 3.6 kV in negative; capillary temperature, 300 °C; sheath gas, 60 L/min; auxiliary gas. 25 L/min; spare gas, 2 L/min; max spray current, 100; probe heater temperature, 370 °C. The instrument was operated in a Top4 data-dependent acquisition (DDA) mode. MS1 spectra were acquired within m/z 60–900 with an automatic gain control (AGC) of 1 × 10^6^ at 60,000 resolution and with a maximum ion time (IT) of 100 ms. Up to 4 of the most abundant precursor ions singly charged were selected for higher-energy C-trap dissociation (HCD). An isolation window of 1 m/z and normalized collision energies of 20, 30 and 40 were used. Ions that were once selected for acquisition were dynamically excluded for 2 s for further fragmentation. MS2 spectra were acquired with an AGC of 1 × 10^5^ at resolution of 15,000. Method blanks and pooled mixtures of all control and treated samples (cells and extracellular medium separately) were included as quality control samples, and were subjected to iterative data acquisition to increase the coverage of the MSMS spectra acquired.

### Charged-surface hybrid chromatography-QExactive mass spectrometry (CSH-QE MS/MS) analysis

The non-polar layers from the cell extracts or the extracellular medium were resuspended in 50 μL of methanol:toluene (9:1, v/v) mixture containing 50 ng/mL of CUDA. 10 μL of cell extract samples were diluted (1:4) with the resuspension solvent to avoid column MS signal saturation. Aliquots of 3 μL (for ESI positive) and 5 μL (for ESI negative) were analyzed using the same LC–MS/MS instrument as for the polar fraction analyses, but compounds were separated using a Waters Acquity CSH C18 column (100 mm length × 2.1 mm id; 1.7 μm particle size) with an additional Waters Acquity VanGuard CSH C18 pre-column (5 mm × 2.1 mm id; 1.7 μm particle size). Columns were kept at 65◦C during the analysis of the samples. Both positive and negative modes used 60:40 v/v ACN:water (LC–MS grade) as mobile phase (A) and 90:10 v/v isopropanol:ACN as mobile phase (B). However, and to improve lipid coverage, different mobile phase modifiers were used for positive (10 mM ammonium formate and 0.1% formic acid) and negative (10 mM ammonium acetate) mode analyses^76^. A flow rate of 0.6 mL/min was used, and the gradient started at 0 min with 15% (B), 0–2 min 30% (B), 2–2.5 min 48% (B), 2.5–11 min 82% (B), 11–1.5 min 99% (B), 11.5–12 min 99% (B), 12–12.1 min 15% (B), and 12.1–15 min 15% (B). The mass spectrometer was operated using the same parameters as for the analysis of the polar fractions, but the mass scan range was adjusted to 200–1700 m/z.

### Quality control

Quality control was assured by: (1) randomization of the sequence; (2) injection of pool samples to equilibrate the LC–MS or GC–MS systems before and after of the different sequence of samples; (3) procedure blank analysis; (4) checking the retention time, the peak shape and the intensity of the spiked internal standards; and (5) monitoring mass accuracy of internal standards during the run.

### Data processing

GM-TOF MS raw data files were exported to mzml format using ChromaTOF-HRT(v 1.74) followed by converting the files to ABF format using Reifycs Abf (Analysis Base File) Converter (accessible at: http://www.reifycs.com/AbfConverter/). LC–MS raw data files were converted to ABF format. Data processing of ABF converted files was performed using MS-DIAL (v. 4.12) software for deconvolution, peak picking, alignment, and identification^77^. For all data sets, *in-house* m/z and retention time libraries were used in addition to public MS/MS spectra databases in MSP format^78,79^. The set of data for each analytical platform were processed separately, as well as the data for cell extracts and the extracellular medium. The following parameters were used for GC–MS data processing: retention time begin, 5 min; retention time end, 20 min; mass range begin, 80 Da; mass range end, 500 Da; smoothing level, 3 scans; average peak width, 20 scans; minimum peak height, 1000 amplitude; mass slice width, 0.05 Da; sigma window value for deconvolution, 0.5; EI spectra cut off, 10 amplitude. Retention index using FAMEs was used with the following parameters: retention index tolerance for MSP library identification, 2000; EI similarity cut off, 70%; identification score cut off and similarity tolerance, 70%. The annotation database used (Fiehn BinBase DB, Rtx5-Sil MS, FAMEs RI) contains 1021 annotated spectra and can be accessed from http://prime.psc.riken.jp/compms/msdial/main.html#MSP. For the data processing of the polar fraction LC–MS analyses, the following parameters were used: retention time begin, 0.1 min; retention time end, 15 min; mass range begin, 60 Da; mass range end, 900 Da; MS1 tolerance, 0.01 Da; smoothing level, 3 scans; minimum peak width, 5 scans; minimum peak height, 10,000 amplitude; mass slice width, 0.5 Da; sigma window value for deconvolution, 0.5; accurate mass tolerance for MSP library, 0.01 Da; identification score cut off for MSP library, 80%; retention time tolerance for retention time-m/z (tR-m/z) library, 0.1 min; accurate mass tolerance for tR-m/z library, 0.01 Da; identification score cut off for tR-m/z library, 85%; retention time tolerance for alignment, 0.1 min; MS1 tolerance for alignment, 0.015 Da. Peak height calculation was performed by combining data for different detected molecular species for each particular compound ([M + H] + , [M + NH4] + , [M + Na] + , [M + K] + adducts in positive mode, and [M-H]-, [M + Cl]-, [M + FA-H]- adducts in negative mode). The following internal standards (CUDA, creatinine-d_3,_ betaine-d_9_, Nτ-methyl-histamine-d_3_, creatine-d_3_, DL-alanine-d_3_, L-glutamine-d_5_, DL-glutamic acid-d_3_, DL-aspartic acid-d_3_, choline-d_9_, TMAO-d_9_, L-carnitine-d_3_, 15N_2_-L-arginine, DL-cystine-d_4_, L-alanine-d_4_, L-arginine-d_7_, L-asparagine-d_3_, L-glutamic acid-d_5_, L-histidine-d_5_, L-isoleucine-d_10_, L-leucine-d_10_, L-lysine-d_8_, L-methionine-d_8_, L-ornithine-d_2_, L-phenylalanine-d_8_, L-proline-d_7_, L-serine-d_3_, L-threonine-d_5_, L-tryptophan-d_8_, L-tyrosine-d_7_, L-valine-d_8_, 1-methylnicotinamide-d_3_, Val-Tyr-Val and acetyl-L-carnitine-d_3_) were used for retention time correction and for compound identification using the tR-m/z library (with 869 and 715 authentic standards with m/z and MS/MS match in positive and negative modes, respectively). The MSP file used for annotation was generated by combining MS/MS spectra from NIST17 MS/MS database, the LipidBLAST mass spectral library^78^ and MassBank of NorthAmerica (MoNA available at http://massbank.us) databases. The parameters used for the data processing of the non-polar fraction LC–MS analyses were: retention time begin, 0.1 min; retention time end, 13 min; mass range begin, 200 Da; mass range end, 1700 Da; MS1 tolerance, 0.01 Da; smoothing level, 3 scans; minimum peak width, 5 scans; minimum peak height, 10,000 amplitude; mass slice width, 0.5 Da; sigma window value for deconvolution, 0.5; accurate mass tolerance for MSP library, 0.01 Da; identification score cut off for MSP library, 80%; retention time tolerance for tR-m/z library, 0.1 min; accurate mass tolerance for tR-m/z library, 0.01 Da; identification score cut off for tR-m/z library, 85%; retention time tolerance for alignment, 0.1 min; MS1 tolerance for alignment, 0.015 Da. For lipid identification, accurate mass and MS/MS matching was used with the public LipidBlast library^78^. Peak height calculation was performed by combining data for different detected molecular species for each particular compound ([M + H] + , [M + NH4] + , [M + Na] + , [M + K] + , [2M + H] + , [2M + NH4] + , [2M + Na] + , [2M + K] + adducts in positive mode, and [M-H]-, [M + Cl]-, [M + FA-H]- adducts in negative mode). The following internal standards (CUDA, LPE (17:1), PI (15:0/18:1)-d_7_, PS (15:0/18:1)-d_7_, LPC (17:0), PC (12:0/13:0), PE (17:0/17:0), PG (17:0/17:0), cholesterol-d_7_, SM (d18:1/17:0), Cer (d18:1/17:0), sphingosine (d17:1), MG (17:0/0:0/0:0), DG (12:0/12:0/0:0), DG (18:1/2:0/0:0), TG (17:0/17:1/17:0)-d_5_, TG (14:0/16:1/14:0)-d_5_, palmitic acid-d_3_, and 5-PAHSA-d_9_) were used for retention time correction and for compound identification using the tR-m/z lipid library (with 306 and 184 verified lipids with m/z and MS/MS match in positive and negative modes, respectively). The MSP file used for annotation was generated by combining MS/MS spectra from NIST17 and LipidBLAST. Metabolite were annotated following the Metabolomics Standard Initiative (MSI) guidelines^80,81^: MSI level 1 for metabolites with precursor m/z, *in-house* mzRT libraries and MS/MS spectral library matching; MSI level 2a for metabolites with precursor m/z and MS/MS spectral library matching, and MSI level 2b for metabolites with precursor m/z and *in-house* mzRT library matching.

### Data post-processing and statistical analysis

The list of metabolites was filtered removing unknown metabolites, metabolites present in less than 50% of samples in each group, metabolites with a maximum height below three times the average height in the blank samples, and metabolites with a maximum height below 3000 units. Thereafter, missing values were imputed by half of the minimum value, and the data were processed using the bioinformatic tool MS-FLO (https://msflo.fiehnlab.ucdavis.edu/#/)^82^. Duplicated metabolites and isotopes were removed, and the height of the different adducts from the same compound were combined. Before any statistical analysis, intracellular metabolite signals were normalized by using the cellular protein content to scale each sample. Then, extracellular and intracellular metabolites were normalized further by “Auto scaling” before PCA and PLS-DA were performed by using MetaboAnalyst 4.0 web-based software^18^. Exported variable importance in projection (VIP) scores were used for evaluation and PLS-DA models were evaluated according to the cross-validation of R^2^, Q^2^ value and permutation test. In addition, univariate analysis using the nonparametric Mann–Whitney U test with FDR correction was performed using Statistica software version 13.3 (TIBCO Software Inc., USA), and metabolites were considered significantly altered when 0.5 > FC > 2 and setting the FDR to 0.05.

### Data visualization, enrichment and pathway analysis

Data matrices from each platform were combined to generate a joint dataset for cell extracts and extracellular medium independently. For those metabolites detected in multiple platforms, data with the highest retention time similarity (from the respective tR-m/z libraries), highest similarity score (from the respective MSP files), highest peak intensity, and/or better peak shape were retained.

For metabolic network mapping, the InChiKey or compound names were imported into the web-based Chemical Translation Service (http://cts.fiehnlab.ucdavis.edu/batch)^83^ to obtain the Kyoto Encyclopedia of Genes and Genomes (KEGG) and PubChem Compound Identifiers (CID), and the simplified molecular-input line-entry system (SMILES) codes for all annotated compounds were obtained from the MSP files or from the PubChem Compound Identifier Exchange service (https://pubchem.ncbi.nlm.nih.gov/idexchange/idexchange.cgi). KEGG reactant pairs and Tanimoto similarity calculations were done using MetaMapp^17^. A threshold of 0.7 Tanimoto score was used to define the similarity cutoff for compound structures. The final network graph was imported into Cytoscape 3.7.2^84^, as well as the results generated in Statistica software. The graphs were visualized using a yED organic layout algorithm in Cytoscape. Chemical similarity enrichment calculations were done using ChemRICH^16^.

Metabolite set enrichment analysis was performed using the Enrichment Analysis module on MetaboAnalyst 4.0^18^. Differential metabolites matching the KEGG database were imported and enrichment analysis against *Homo sapiens* pathway-associated metabolite sets was performed. In addition, the Joint Pathway Analysis module was used to integrate the significantly altered metabolites from this study with the proteomics results obtained from our previous study and conducted under the same experimental conditions^26^. For this analysis, hypergeometric tests were used, and p-values were adjusted using the Holm-Bonferroni correction. Metabolic pathways with adjusted p-values lower than 0.05 were considered significantly enriched.

## Supplementary Information

Supplementary Information 1.

Supplementary Information 2.

Supplementary Information 3.

Supplementary Information 4.

Supplementary Information 5.

Supplementary Information 6.

## References

[1] Schena FP, Gesualdo L (2005). Pathogenetic mechanisms of diabetic nephropathy. J. Am. Soc. Nephrol..

[2] Zhang G, Darshi M, Sharma K (2018). The warburg effect in diabetic kidney disease. Semin. Nephrol..

[3] Lim AKH (2014). Diabetic nephropathy—complications and treatment. Int. J. Nephrol. Renovasc. Dis..

[4] Vallon V, Komers R (2011). Pathophysiology of the diabetic kidney. Comp. Physiol..

[5] Abbiss, H., Maker, G. L. & Trengove, R. D. Metabolomics approaches for the diagnosis and understanding of kidney diseases. *Metabolites* **9**, (2019). 10.3390/metabo9020034 PMC6410198 30769897

[6] van der Kloet FM (2012). Discovery of early-stage biomarkers for diabetic kidney disease using ms-based metabolomics (FinnDiane study). Metabolomics.

[7] Sharma K (2013). Metabolomics reveals signature of mitochondrial dysfunction in diabetic kidney disease. J. Am. Soc. Nephrol..

[8] Mäkinen VP (2012). Sphingomyelin is associated with kidney disease in type 1 diabetes (The FinnDiane Study). Metabolomics.

[9] Mäkinen VP (2013). Triglyceride-cholesterol imbalance across lipoprotein subclasses predicts diabetic kidney disease and mortality in type 1 diabetes: The FinnDiane Study. J. Intern. Med..

[10] Barrios C (2018). Circulating metabolic biomarkers of renal function in diabetic and non-diabetic populations. Sci. Rep..

[11] Zhao T (2012). Intrarenal metabolomics reveals the association of local organic toxins with the progression of diabetic kidney disease. J. Pharm. Biomed. Anal..

[12] You YH, Quach T, Saito R, Pham J, Sharma K (2016). Metabolomics reveals a key role for fumarate in mediating the effects of NADPH oxidase 4 in diabetic kidney disease. J. Am. Soc. Nephrol..

[13] Wei T (2015). Metabonomic analysis of potential biomarkers and drug targets involved in diabetic nephropathy mice. Sci. Rep..

[14] Zhang A, Sun H, Wang P, Han Y, Wang X (2012). Modern analytical techniques in metabolomics analysis. Analyst.

[15] Kind T (2018). Identification of small molecules using accurate mass MS/MS search. Mass Spectrom. Rev..

[16] Barupal DK, Fiehn O (2017). Chemical similarity enrichment analysis (ChemRICH) as alternative to biochemical pathway mapping for metabolomic datasets. Sci. Rep..

[17] Barupal DK (2012). MetaMapp: mapping and visualizing metabolomic data by integrating information from biochemical pathways and chemical and mass spectral similarity. BMC Bioinform..

[18] Chong J, Wishart DS, Xia J (2019). Using MetaboAnalyst 4.0 for comprehensive and integrative metabolomics data analysis. Curr. Protoc. Bioinforma.

[19] Vallon, V. The proximal tubule in the pathophysiology of the diabetic kidney. *Am. J. Physiol. - Regul. Integr. Comp. Physiol.* **300**, (2011). 10.1152/ajpregu.00809.2010 PMC3094037 21228342

[20] Slyne J, Slattery C, McMorrow T, Ryan MP (2015). New developments concerning the proximal tubule in diabetic nephropathy: in vitro models and mechanisms. Nephrol. Dial. Transplant..

[21] Wei PZ (2019). Metabolomic changes of human proximal tubular cell line in high glucose environment. Sci. Rep..

[22] Bernardo-Bermejo S (2020). A non-targeted capillary electrophoresis-mass spectrometry strategy to study metabolic differences in an in vitro model of high-glucose induced. Molecules.

[23] Bernardo-Bermejo S (2019). An untargeted metabolomic strategy based on liquid chromatography-mass spectrometry to study high glucose-induced changes in HK-2 cells. J. Chromatogr. A.

[24] Catrina SB (2014). Impaired hypoxia-inducible factor (HIF) regulation by hyperglycemia. J. Mol. Med..

[25] García-Pastor C, Benito-Martínez S, Moreno-Manzano V, Fernández-Martínez AB, Lucio-Cazaña FJ (2019). Mechanism and consequences of the impaired Hif-1α response to hypoxia in human proximal tubular HK-2 cells exposed to high glucose. Sci. Rep..

[26] Valdés A, Castro-Puyana M, García-Pastor C, Lucio-Cazaña FJ, Marina ML (2020). Time-series proteomic study of the response of HK-2 cells to hyperglycemic, hypoxic diabetic-like milieu. PLoS ONE.

[27] Okuda S (2008). KEGG Atlas mapping for global analysis of metabolic pathways. Nucleic Acids Res..

[28] Mandel LJ (1985). Metabolic substrates, cellular energy production, and the regulation of proximal tubular transport. Annu. Rev. Physiol..

[29] Hato T (2016). Novel application of complementary imaging techniques to examine in vivo glucose metabolism in the kidney. Am. J. Physiol. Renal. Physiol..

[30] Elhamri M, Martin M, Ferrier B, Baverel G (1993). Substrate uptake and utilization by the kidney of fed and starved rats in vivo. Ren. Physiol. Biochem..

[31] Li L (2019). FXR activation alleviates tacrolimus-induced post-transplant diabetes mellitus by regulating renal gluconeogenesis and glucose uptake. J. Transl. Med..

[32] Sasaki M (2017). Dual regulation of gluconeogenesis by insulin and glucose in the proximal tubules of the kidney. Diabetes.

[33] Zager RA, Johnson AC, Hanson SY (2003). Proximal tubular cholesterol loading after mitochondrial, but not glycolytic, blockade. Am. J. Physiol. Renal Physiol..

[34] Czajka A, Malik AN (2016). Hyperglycemia induced damage to mitochondrial respiration in renal mesangial and tubular cells: Implications for diabetic nephropathy. Redox Biol..

[35] Sas, K. M. et al. Tissue-specific metabolic reprogramming drives nutrient flux in diabetic complications. *JCI Insight.* **1**, (2016). 10.1172/jci.insight.86976 PMC5033761 27699244

[36] Kim NH (2018). Metabolic changes in urine and serum during progression of diabetic kidney disease in a mouse model. Arch. Biochem. Biophys..

[37] Maitre M (1997). The γ-hydroxybutyrate signalling system in brain: Organization and functional implications. Prog. Neurobiol..

[38] Bhargava P, Schnellmann RG (2017). Mitochondrial energetics in the kidney. Nat. Rev. Nephrol..

[39] Dunlop M (2000). Aldose reductase and the role of the polyol pathway in diabetic nephropathy. Kidney Int. Suppl..

[40] Chevrollier A, Loiseau D, Reynier P, Stepien G (2011). Adenine nucleotide translocase 2 is a key mitochondrial protein in cancer metabolism. Biochim. Biophys. Acta Bioenerg..

[41] Guan SS, Sheu ML, Wu CT, Chiang CK, Liu SH (2015). ATP synthase subunit-β down-regulation aggravates diabetic nephropathy. Sci. Rep..

[42] Yan L (2018). Redox imbalance stress in diabetes mellitus: role of the polyol pathway. Anim. Model. Exp. Med..

[43] Wu M (2019). Mitochondrial activity contributes to impaired renal metabolic homeostasis and renal pathology in STZ-induced diabetic mice. Am. J. Physiol. Ren. Physiol..

[44] Pcal L (2011). Role of thiamine status and genetic variability in transketolase and other pentose phosphate cycle enzymes in the progression of diabetic nephropathy. Nephrol. Dial. Transplant..

[45] Sharma V, Ichikawa M, Freeze HH (2014). Mannose metabolism: more than meets the eye. Biochem. Biophys. Res. Commun..

[46] Noh H, King GL (2007). The role of protein kinase C activation in diabetic nephropathy. Kidney Int..

[47] Geraldes P, King GL (2010). Emission security-tempest attacks. Circ. Res..

[48] Temes E (2004). Role of diacylglycerol induced by hypoxia in the regulation of HIF-1a activity. Biochem. Biophys. Res. Commun..

[49] Popolo A, Adesso S, Pinto A, Autore G, Marzocco S (2014). L-Arginine and its metabolites in kidney and cardiovascular disease. Amino Acids.

[50] Wever R (1999). Nitric oxide production is reduced in patients with chronic renal failure. Arterioscler. Thromb. Vasc. Biol..

[51] Satriano J, Kelly CJ, Blantz RC (1999). An emerging role for agmatine. Kidney Int..

[52] Li G (1994). Agmatine: an endogenous clonidine-displacing substance in the brain. Science.

[53] Marx M, Trittenwein G, Aufricht C, Hoeger H, Lubec B (1995). Agmatine and spermidine reduce collagen accumulation in kidneys of diabetic db/db mice. Nephron.

[54] Albrecht T (2017). Carnosine attenuates the development of both type 2 diabetes and diabetic nephropathy in BTBR ob/ob mice. Sci. Rep..

[55] Janssen B (2005). Carnosine as a protective factor in diabetic. Diabetes.

[56] Gallazzini M, Burg MB (2009). What’s new about osmotic regulation of glycerophosphocholine. Physiology.

[57] Ohvo-Rekilä H, Ramstedt B, Leppimäki P, Peter Slotte J (2002). Cholesterol interactions with phospholipids in membranes. Prog. Lipid Res..

[58] van der Veen JN (2017). The critical role of phosphatidylcholine and phosphatidylethanolamine metabolism in health and disease. Biochim. Biophys. Acta Biomembr..

[59] Ueda N (2001). Role of ceramide synthase in oxidant injury to renal tubular epithelial cells. J. Am. Soc. Nephrol..

[60] Basnakian AG (2005). Ceramide synthase is essential for endonuclease-mediated death of renal tubular epithelial cells induced by hypoxia-reoxygenation. Am. J. Physiol. Ren. Physiol..

[61] Zager RA, Burkhart KM, Johnson ACM, Sacks BM (1999). Increased proximal tubular cholesterol content: Implications for cell injury and ‘acquired cytoresistance’. Kidney Int..

[62] Warner GJ, Stoudt G, Bamberger M, Johnson WJ, Rothblat GH (1995). Cell toxicity induced by inhibition of acyl coenzyme A: cholesterol acyltransferase and accumulation of unesterified cholesterol. J. Biol. Chem..

[63] Zager RA, Kalhorn TF (2000). Changes in free and esterified cholesterol: Hallmarks of acute renal tubular injury and acquired cytoresistance. Am. J. Pathol..

[64] Sanchez-Niño MD, Benito-Martin A, Ortiz A (2010). New paradigms in cell death in human diabetic nephropathy. Kidney Int..

[65] McCarty MF, DiNicolantonio JJ (2014). β-Alanine and orotate as supplements for cardiac protection. Open Heart..

[66] Bene J, Hadzsiev K, Melegh B (2018). Role of carnitine and its derivatives in the development and management of type 2 diabetes. Nutr. Diabetes.

[67] Barlow JP (2020). Beta-aminoisobutyric acid is released by contracting human skeletal muscle and lowers insulin release from INS-1 832/3 cells by mediating mitochondrial energy metabolism. Metab. Open.

[68] Sánchez-Romero N, Schophuizen CM, Giménez I, Masereeuw R (2016). In vitro systems to study nephropharmacology: 2D versus 3D models. Eur. J. Pharmacol..

[69] Lee JW, Chou CL, Knepper MA (2015). Deep sequencing in microdissected renal tubules identifies nephron segment-specific transcriptomes. J. Am. Soc. Nephrol..

[70] Jenkinson SE, Chung GW, van Loon E, Bakar NS, Dalzell AM, Brown CD (2012). The limitations of renal epithelial cell line HK-2 as a model of drug transporter expression and function in the proximal tubule. Pflugers Arch..

[71] Yánez AJ (2003). Broad expression of fructose-1,6-bisphosphatase and phosphoenolpyruvate carboxykinase provide evidence for gluconeogenesis in human tissues other than liver and kidney. J. Cell Physiol..

[72] Di Mise A (2015). Conditionally immortalized human proximal tubular epithelial cells isolated from the urine of a healthy subject express functional calcium-sensing receptor. Am. J. Physiol. Renal Physiol..

[73] Rahmoune H, Thompson PW, Ward JM, Smith CD, Hong G, Brown J (2005). Glucose transporters in human renal proximal tubular cells isolated from the urine of patients with non-insulin-dependent diabetes. Diabetes.

[74] Matyash V, Liebisch G, Kurzchalia TV, Shevchenko A, Schwudke D (2008). Lipid extraction by methyl-terf-butyl ether for high-throughput lipidomics. J. Lipid Res..

[75] Megan R (2018). Obesogenic diets alter metabolism in mice. PLoS ONE.

[76] Cajka T, Fiehn O (2016). Increasing lipidomic coverage by selecting optimal mobile-phase modifiers in LC-MS of blood plasma. Metabolomics.

[77] Tsugawa H (2015). MS-DIAL: data-independent MS/MS deconvolution for comprehensive metabolome analysis. Nat. Methods.

[78] Kind T (2013). LipidBlast in silico tandem mass spectrometry database for lipid identification. Nat. Methods.

[79] Blaženović I (2019). Structure annotation of all mass spectra in untargeted metabolomics. Anal. Chem..

[80] Sumner LW (2007). Proposed minimum reporting standards for chemical analysis: chemical analysis working group (CAWG) metabolomics standards initiative (MSI). Metabolomics.

[81] Blaženović, I., Kind, T., Ji, J. & Fiehn, O. Software tools and approaches for compound identification of LC-MS/MS data in metabolomics. *Metabolites* **8**, (2018). 10.3390/metabo8020031 PMC6027441 29748461

[82] DeFelice BC (2017). Mass spectral feature list optimizer (MS-FLO): a tool to minimize false positive peak reports in untargeted liquid chromatography-mass spectroscopy (LC-MS) data processing. Anal. Chem..

[83] Wohlgemuth G, Haldiya PK, Willighagen E, Kind T, Fiehn O (2010). The chemical translation service—a web-based tool to improve standardization of metabolomic reports. Bioinformatics.

[84] Shannon P (2003). Cytoscape: a software environment for integrated models of biomolecular interaction networks. Genome Res..

