# Supplementary material for: Comprehensive metabolomic study of the response of HK-2 cells to hyperglycemic hypoxic diabetic-like milieu
Source: Sci Rep. 2021 Mar 3;11:5058. doi: 10.1038/s41598-021-84590-2 (PMC7930035; doi:10.1038/s41598-021-84590-2)

Supplementary Information for

Comprehensive metabolomic study of the response of HK-2 cells to hyperglycemic hypoxic diabetic-like milieu

Alberto Valdés[1,2]*, Francisco J. Lucio-Cazaña[3], María Castro-Puyana[1,4], Coral García-Pastor[3], Oliver Fiehn[2], María Luisa Marina[1,4]*

[1]Departamento de Química Analítica, Química Física e Ingeniería Química, Universidad de Alcalá, Ctra. Madrid-Barcelona, Km. 33.600, 28871 Alcalá de Henares, Madrid, España.

[2]West Coast Metabolomics Center, UC Davis, Davis, CA, United States.

[3]Departamento de Biología de Sistemas, Universidad de Alcalá, Ctra. Madrid-Barcelona, Km. 33.600, 28871 Alcalá de Henares, Madrid, España.

[4]Instituto de Investigación Química Andrés M del Rio, IQAR, Universidad de Alcalá, Ctra. Madrid-Barcelona, Km. 33.600, 28871 Alcalá de Henares, Madrid, España.

* Corresponding authors:

avaldes@ucdavis.edu (AV); ORCID: 0000-0002-7901-5816
mluisa.marina@uah.es (MLM); ORCID: 0000-0002-5583-1624

Table of contents

Supplementary Figure S1. Scatterplots of retention time (**A**) and fold change values (**B**) of commonly annotated intracellular metabolites by HILIC-QE MS/MS (+) and HILIC-QE MS/MS (-). Scatterplots of retention time (**C**) and fold change values (**D**) of commonly annotated intracellular metabolites by CSH-QE MS/MS (+) and CSH-QE MS/MS (-). The correlations are presented by Pearson's correlation coefficients (r).

Supplementary Figure S2. Scatterplots of retention time (**A**) and fold change values (**B**) of commonly annotated extracellular metabolites by HILIC-QE MS/MS (+) and HILIC-QE MS/MS (-). The correlations are presented by Pearson's correlation coefficients (r).

Supplementary Figure S3. PCA score plots of data obtained by GC-TOF MS, HILIC-QE MS/MS (+), HILIC-QE MS/MS (-), CSH-QE MS/MS (+) and CSH-QE MS/MS (-) from the intracellular medium after incubation of HK-2 cells in HG (25 mM glucose)-hypoxia (1% O_2) compared to control conditions (5.5 mM glucose/18.6% O_2) for 48 h.

Supplementary Figure S4. PLS-DA score plots of data obtained by GC-TOF MS, HILIC-QE MS/MS (+), HILIC-QE MS/MS (-), CSH-QE MS/MS (+) and CSH-QE MS/MS (-) from the intracellular medium after incubation of HK-2 cells in HG (25 mM glucose)-hypoxia (1% O_2) compared to control conditions (5.5 mM glucose/18.6% O_2) for 48 h.

Supplementary Figure S5. PCA score plots of data obtained by GC-TOF MS, HILIC-QE MS/MS (+), HILIC-QE MS/MS (-), CSH-QE MS/MS (+) and CSH-QE MS/MS (-) from the extracellular medium after incubation of HK-2 cells in HG (25 mM glucose)-hypoxia (1% O_2) compared to control conditions (5.5 mM glucose/18.6% O_2) for 48 h.

Supplementary Figure S6. PLS-DA score plots of data obtained by GC-TOF MS, HILIC-QE MS/MS (+), HILIC-QE MS/MS (-), CSH-QE MS/MS (+) and CSH-QE MS/MS (-) from the extracellular medium after incubation of HK-2 cells in HG (25 mM glucose)-hypoxia (1% O_2) compared to control conditions (5.5 mM glucose/18.6% O_2) for 48 h.

Supplementary Table S1. Relative standard deviation of the labelled internal standards included during sample preparation and obtained from each analytical platform.

Supplementary Table S2. Lists of annotated metabolites in each analytical platform and ionization modes in the intracellular medium after incubation of HK-2 cells in HG (25 mM glucose)-hypoxia (1% O_2) compared to control conditions (5.5 mM glucose/18.6% O_2) for 48 h.

Supplementary Table S3. Lists of annotated metabolites in each analytical platform and ionization modes in the extracellular medium after incubation of HK-2 cells in HG (25 mM glucose)-hypoxia (1% O_2) compared to control conditions (5.5 mM glucose/18.6% O_2) for 48 h.

Supplementary Table S4. List of significantly altered metabolites (combining different analytical platforms and ionization modes) in the intracellular and extracellular medium after incubation of HK-2 cells in HG (25 mM glucose)-hypoxia (1% O_2) compared to control conditions (5.5 mM glucose/18.6% O_2) for 48 h.

Supplementary Table S5. Concentration of internal standards used in GC-TOF MS, CSH-QE MS/MS and HILIC-QE MS/MS analyses.

Supplementary Figure S1.

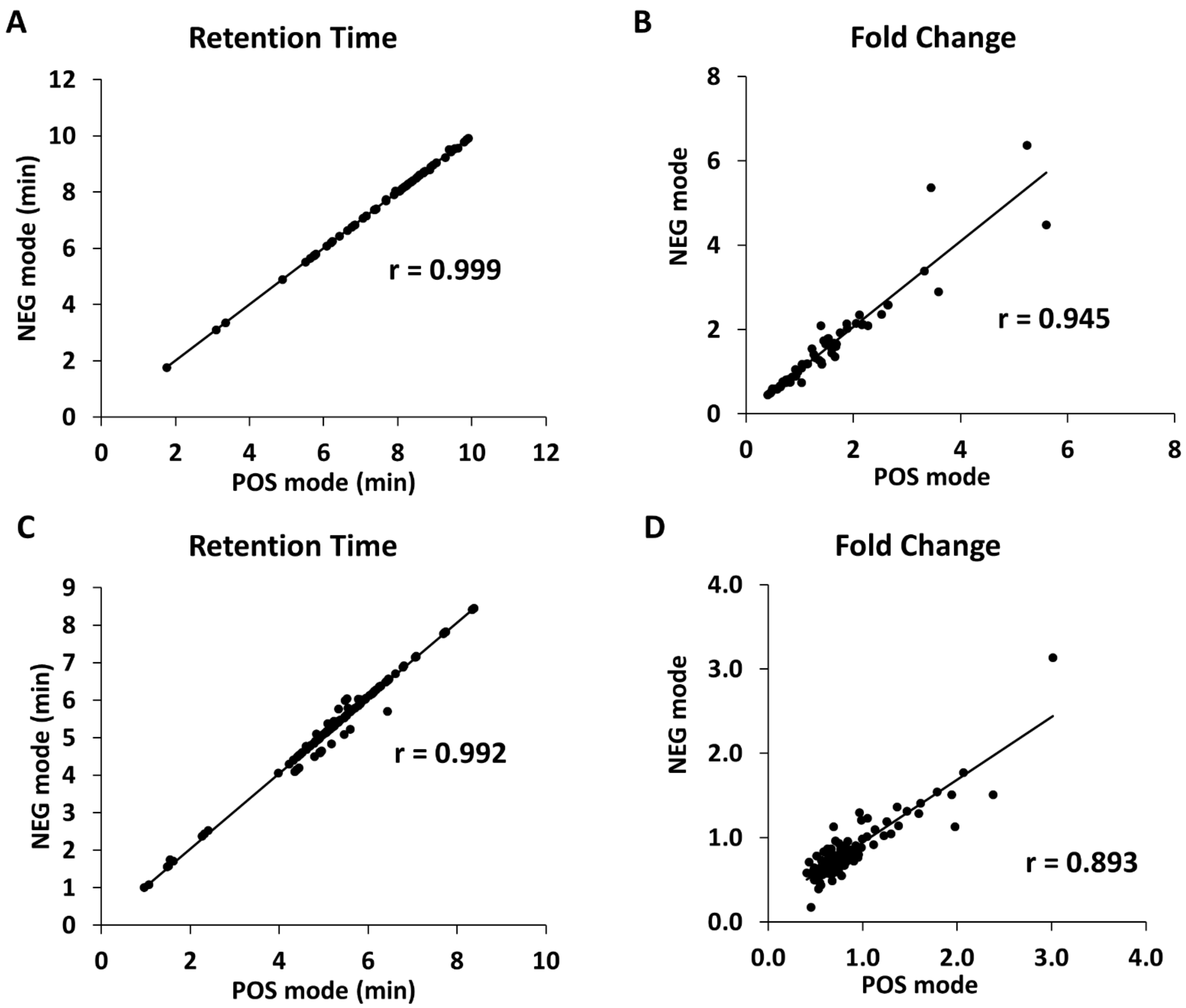

Supplementary Figure S2.

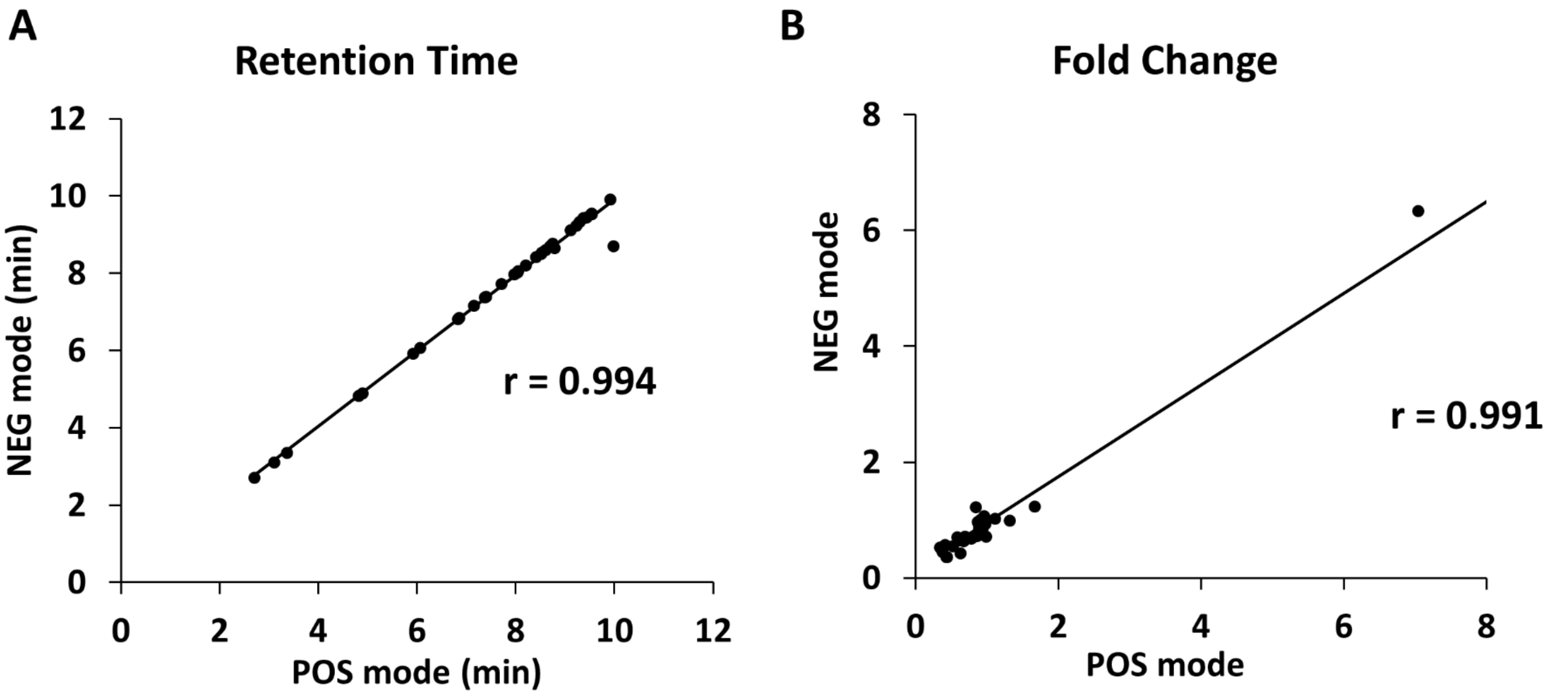

Supplementary Figure S3.

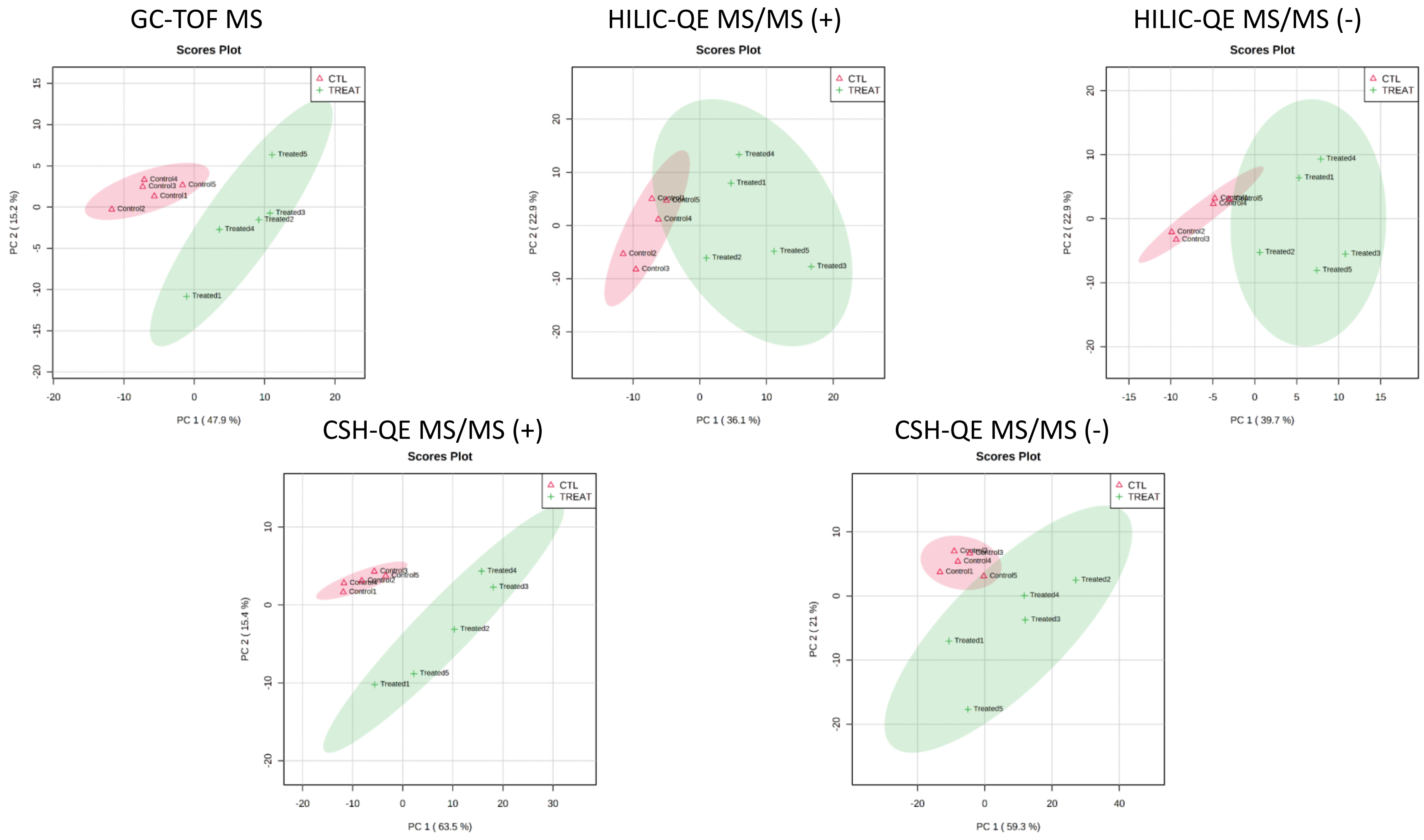

Supplementary Figure S4.

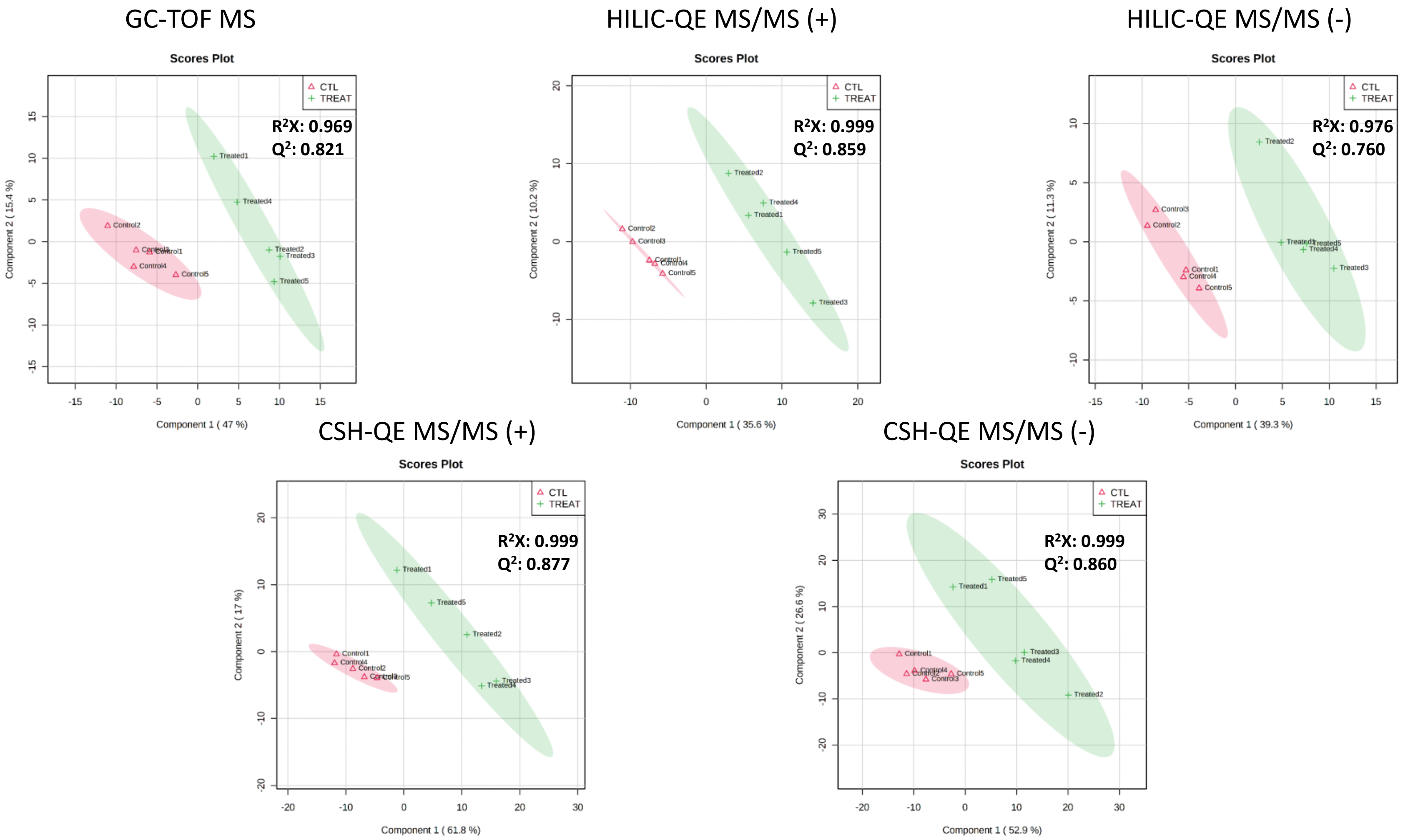

Supplementary Figure S5.

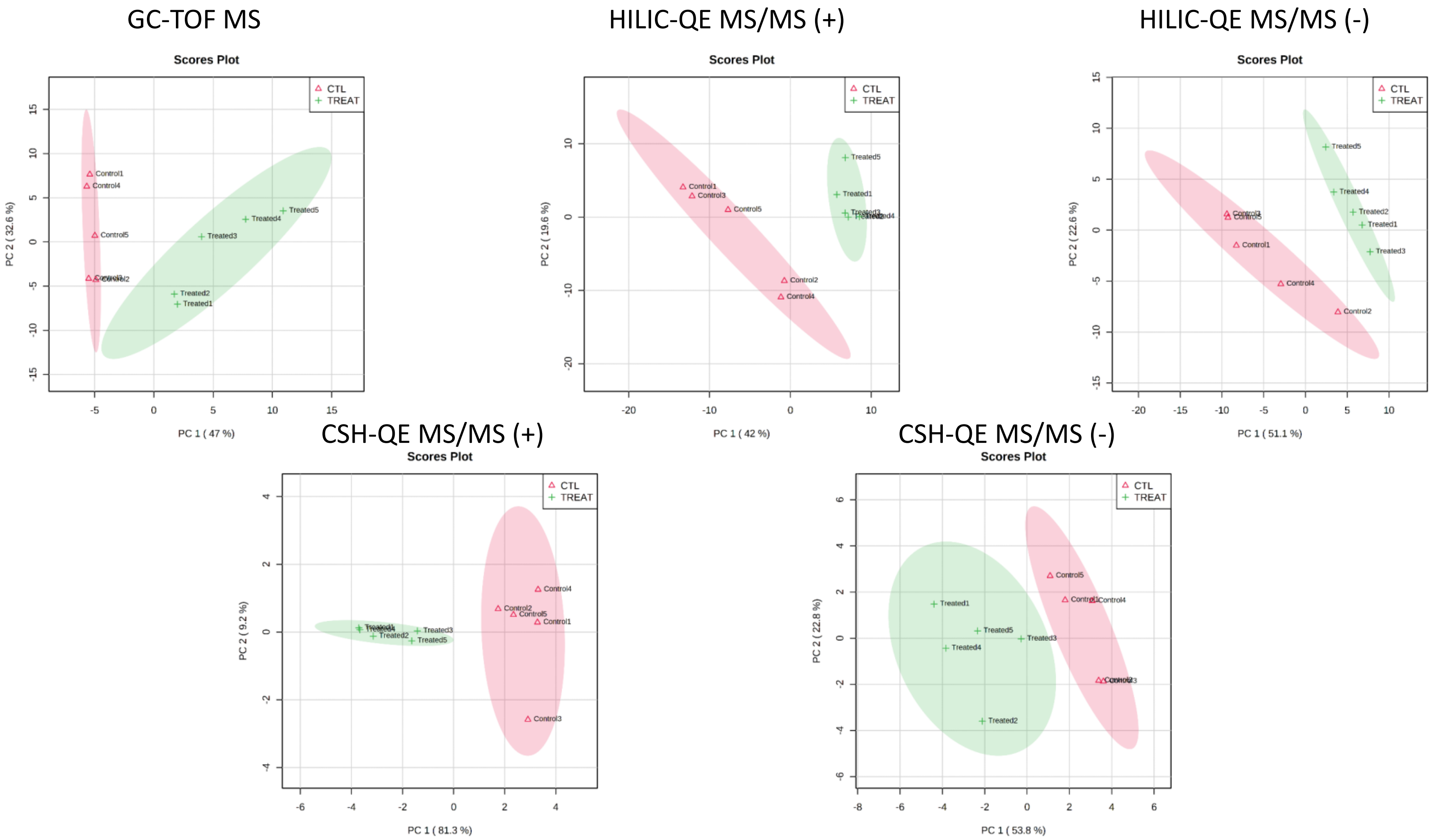

Supplementary Figure S6.

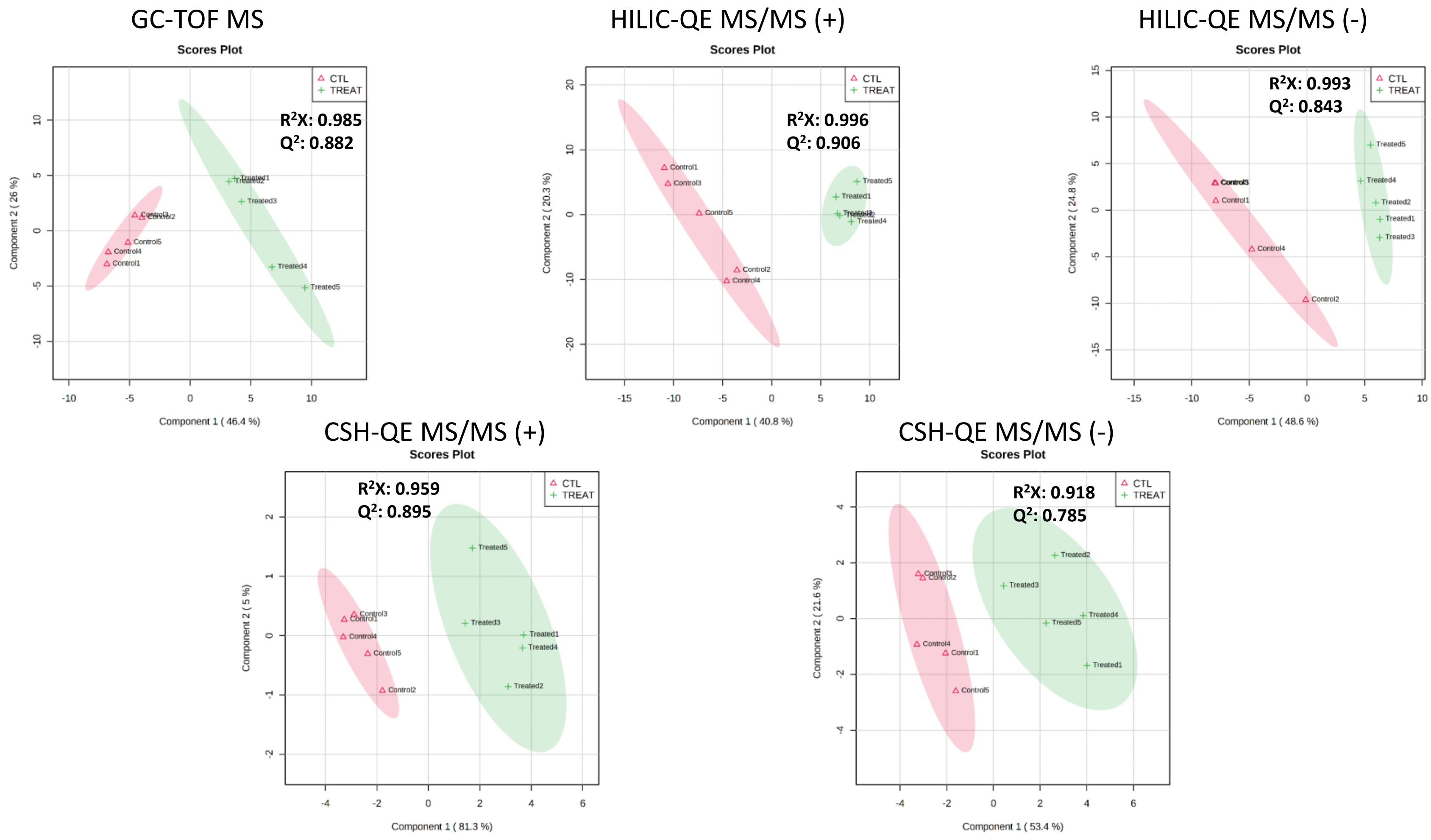

Supplement: Supplementary file 1 — Supplementary Information 1. [file 41598_2021_84590_MOESM1_ESM.pdf]
